# Adverse events during oral colchicine use: a systematic review and meta-analysis of randomised controlled trials

**DOI:** 10.1186/s13075-020-2120-7

**Published:** 2020-02-13

**Authors:** Sarah Stewart, Kevin Chih Kai Yang, Kate Atkins, Nicola Dalbeth, Philip C. Robinson

**Affiliations:** 1 Bone & Joint Research Group, Faculty of Medicine, University of Auckland, Auckland, New Zealand; 2 School of Clinical Medicine, Faculty of Medicine, University of Queensland, Brisbane, Australia; 3 Department of Rheumatology, Royal Brisbane Hospital, Bowen Bridge Road, Herston, QLD 4006 Australia

**Keywords:** Colchicine, Gout, Diarrhoea, Nausea

## Abstract

**Background:**

Colchicine is a widely used drug to treat inflammatory diseases. Due to its long historical use in medicine, controlled clinical trials have been small and there remains some caution with the use of this drug in patients with co-morbidities. The aim of the study is to systematically examine the side effect profile of colchicine in controlled clinical trials across all published indications.

**Methods:**

A systematic review was conducted in accordance with PRISMA methodology. The Cochrane Library, MEDLINE and EMBASE were searched for double-blind controlled trials of oral colchicine in adult patients that reported adverse event data. Meta-analyses were used to determine the relative risk (RR) of adverse events in colchicine users compared to comparator groups.

**Results:**

A total of 4915 studies were initially identified and after exclusions, 35 randomised controlled trials with placebo (*n* = 35) or active comparators (*n* = 5) were included. The most common diseases studied were gout, liver cirrhosis and pericarditis. There were a total of 8659 pooled participants, 4225 participants were randomised to receive colchicine, 3956 to placebo and 411 to an active comparator. Diarrhoea was reported in 17.9% of colchicine users versus 13.1% in comparator groups (RR 2.4, 95% confidence interval (CI) 1.6, 3.7). Any gastrointestinal event was reported in 17.6% of colchicine users and 13.1% of comparators (RR 1.7, 95% CI 1.3, 2.3). Adverse liver events were reported in 1.9% of colchicine users versus 1.1% in the comparator groups (RR 1.6, 95% CI 0.9, 3.0). Muscle events were reported in 4.2% of colchicine users and 3.3% in the comparator groups (RR 1.3, 95% CI 0.8, 1.9). Haematology events were reported in 0.6% of colchicine users and 0.4% of comparator groups (RR 1.34 (0.64, 2.82). No study reported neuropathy events. Other sensory events were reported in 1.1% of colchicine users and 1.5% of comparator groups (RR 1.4, 95% CI 0.3, 6.7). Infectious events were reported in 0.4% of colchicine users and 2.1% of comparator groups (RR 1.0, 95% CI 0.7, 1.5). No study reported death as an adverse event.

**Conclusion:**

Colchicine increases the rate of diarrhoea and gastrointestinal adverse events but does not increase the rate of liver, sensory, muscle, infectious or haematology adverse events or death.

## Introduction

Colchicine is an anti-inflammatory agent which is widely used for the treatment of gout and also used extensively for familial Mediterranean fever, Behcet’s disease and pericarditis. Its use in the management of gout has increased due to the widespread recommendation that it be used as a gout flare prophylaxis when urate-lowering therapy is initiated [1]. It is used continuously for long periods of time in individuals with familial Mediterranean fever and Behcet’s disease. However, due to its long historical use in medicine, it has not been subjected to the same registration trials that contemporary medicines require. There remains uncertainty regarding its use in certain risk groups including those with kidney and liver impairment, at higher doses, and with CYP3A4 inhibitors [2]. It has previously been used in an intravenous preparation, but this is no longer used due to the adverse safety profile of this administration method [3].

Although the adverse event profile of colchicine has been reported in various individual clinical trials and for single indications like pericarditis [4], it has not been studied systematically to our knowledge. The aim of this study was to examine the adverse events of colchicine reported in randomised controlled trials using a systemic review and meta-analysis methodology.

## Methods

This study was conducted according to a pre-defined protocol using the Preferred Reporting Items for Systematic Reviews and Meta-Analyses (PRISMA) statement [5].

### Search strategy

Electronic databases (Cochrane Library, MEDLINE and EMBASE) were searched from inception to November 2019 using the following key words in the search term: colchicine AND ((randomised controlled trial [pt] OR controlled clinical trial [pt] OR randomised [tiab] OR placebo [tiab] OR drug therapy [sh] OR randomly [tiab] OR trial [tiab] OR groups [tiab]) NOT (animals [mh] NOT humans [mh])). Bibliographical references from individual included studies and review articles were also hand-searched to identify additional relevant papers. All studies generated from the search were exported to RefWorks and screened to remove duplicates. Title and abstract screening, followed by full-text screening, was undertaken by a single reviewer (KA). Randomised controlled trials were included if they compared the effects of colchicine in patients, administered for any indication, to placebo or active comparators. Trials were included if they: had a double-blinded design, included oral colchicine in at least one of the treatment arms; involved adult participants; were published in the English language; and reported adverse event data in both the colchicine and comparator group(s) in relation to the number of participants with adverse events per group. Studies which reported the number of participants with adverse events resulting in study withdrawals and did not report the number of adverse events which occurred in the participants who remained in the study, were excluded. There was no publication date restriction. If multiple reports described the same trial, the most recent full-text publication was selected for inclusion.

### Quality assessment

Quality of all included studies was assessed independently by two reviewers (SS, KY) using the 6-item modified-Jadad scale which assesses reported randomisation, blinding, withdrawals, dropouts, inclusion/exclusion criteria, adverse events and the statistical analysis [6]. The scale has a maximum score of 8 points, with low quality studies yielding scores of 0 to 3 and high-quality studies yielding a score of 4 to 8. Any disagreements in the quality assessment were resolved by discussion of the two reviewers. If necessary, a third reviewer (ND) was involved to reach consensus.

### Data extraction

Two reviewers (SS, KY) independently extracted data from the full-text studies using a Microsoft Excel extraction form. Any disagreements were resolved by consensus with a third reviewer (ND). Data extraction included publication details (author, year of publication, country of first author), disease state, participant characteristics (sample size, ethnicity, mean (SD) age and n % of male participants) and details of the trial (study design, length of follow up, primary outcome, interventions and dosages, intervention length). Extraction of data related to adverse events included the total number of participants with any adverse event per group and the total number of participants with each individual reported adverse event. Data for the number of deaths were extracted only if death was related to an adverse event (rather than worsening of disease).

### Data-analyses

For the purpose of data analysis, adverse events were grouped under eight pre-defined categories: diarrhoea, gastrointestinal events (including diarrhoea), liver events, haematology events, muscle-related events, sensory-related events (including neuropathy), infectious events, death and any adverse event. In situations when studies reported the number of participants with ≥ 2 individual adverse events which both came under the same category (i.e. “nausea” and “vomiting” which both come under the gastrointestinal event category), data from the adverse event with the highest number of participants was used for that category. Only studies which reported diarrhoea as a separate event were included in the diarrhoea category. Studies which reported diarrhoea as part of a combined event (i.e. ‘diarrhoea or nausea’) were included under the ‘gastrointestinal events’ category.

Meta-analyses were undertaken to determine the relative risk of adverse events in the colchicine group compared to the comparator groups (pooled comparators, placebo and active-comparators). Relative risk was calculated based on the number of participants with adverse events. Random effects models were used for all *I*^2^ values > 0%. As caution is recommended when colchicine is used continuously in those with liver impairment, a sensitivity analysis was undertaken excluding the studies involving participants with cirrhosis or sclerosing cholangitis [6–11]. For the ‘any adverse events’ category, meta-analyses were also used to determine the effects of disease indication (liver diseases, gout, Behcet’s and related conditions, pericarditis and related conditions and other), duration of exposure to the intervention (with subgroups defined as ≤ 2 weeks, 1 to 2 months, 3 to 5 months, 6 to 12 months and ≥ 24 months), the average daily dose of colchicine (with subgroups defined as < 1 mg, 1 mg, > 1 < 2 mg, ≥ 2 mg) and the cumulative daily dose of colchicine (with subgroups defined as < 50 mg, 50 to < 100 mg, ≥ 100 mg to 300 mg, > 600 mg). In papers which used different colchicine doses based on participant weight categories, the highest daily colchicine dose was used to determine that study’s subgroup. Subgroup comparisons were made using the Phet statistic (the *P* value derived from the chi-square test of heterogeneity for subgroup differences).

All meta-analyses were undertaken in Review Manager 5.3 with an alpha level of 0.05. Only studies specifically reporting an adverse event as being present or absent were included in the meta-analyses. However, as this may over- or under-estimate the true occurrence of adverse events, the proportion of participants with specific adverse events was also computed in relation to the total number of participants included in all studies (i.e. if not reported, ‘0’ events were considered to have occurred). These data were used for descriptive purposes only and not meta-analysed.

## Results

### Study characteristics

**Fig. 1:**
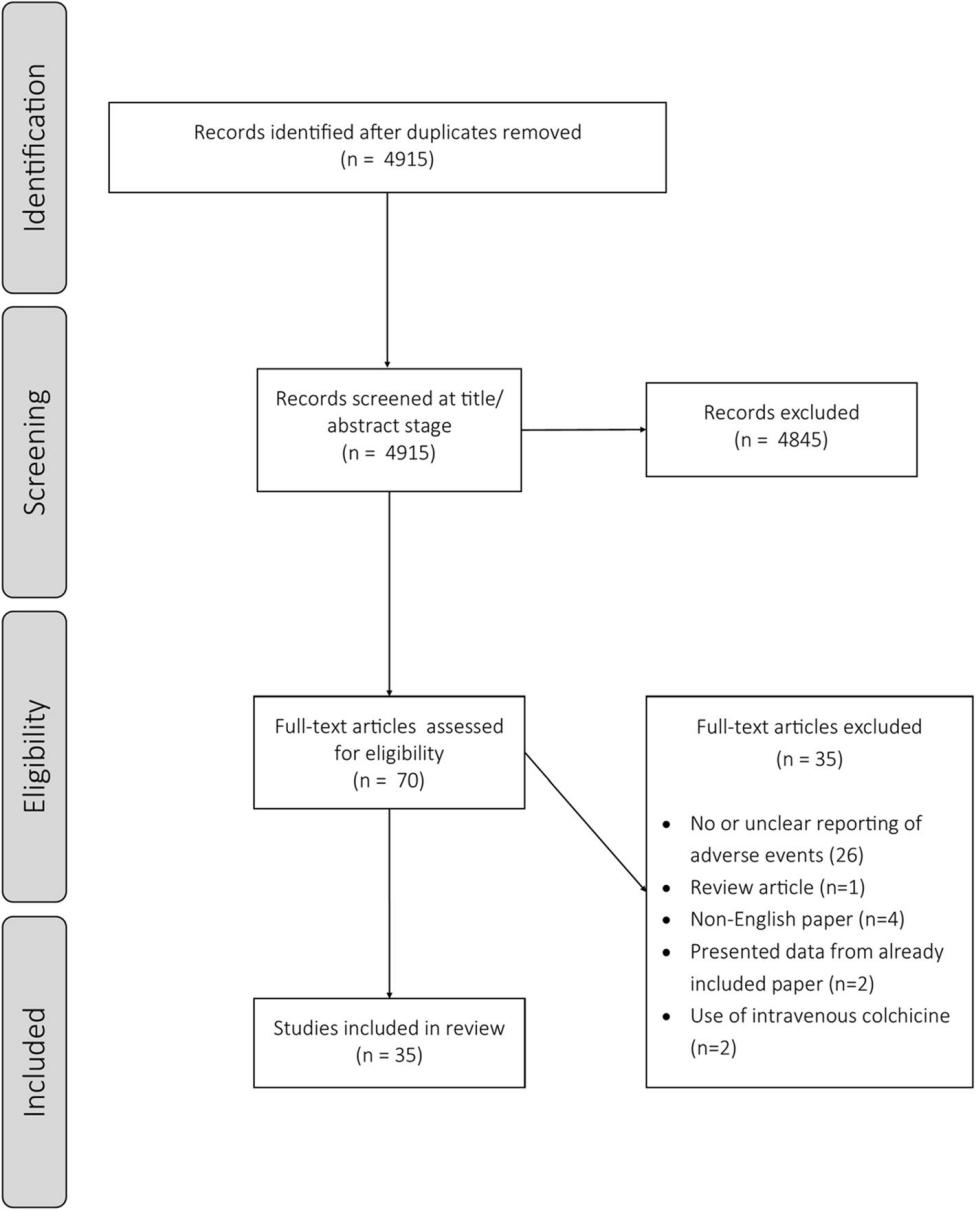
PRISMA flow chart

A total of 4915 studies were identified through the search following the deletion of duplicates (Fig. 1). After title and abstract screening, 70 full-text articles were assessed for eligibility. After the exclusion of 35 studies (reasons for exclusion are presented in Fig. 1), a total of 35 randomised-controlled double-blind studies were included in this review. The majority of studies were placebo-controlled (*n* = 30, 83%) and 5 (17%) studies were active-comparator controlled. The majority of studies were parallel-group designs and 4 studies [12–15] were cross-over designs.

### Quality assessment

The results from the quality assessment are presented in Supplementary Figure 1. Overall, the modified-Jadad score indicated high quality (total score 4 to 8) for all studies. An appropriate method of randomisation and blinding was reported in 24 (69%) and 28 (80%) studies, respectively. Thirty (86%) studies provided an adequate description of withdrawals and dropouts and 33 (94%) provided a participant inclusion and exclusion criteria. The method used to assess adverse events was described by 22 (63%) studies and methods of statistical analysis by 32 (91%).

### Participant characteristics

Characteristics of participants in the included studies are shown in Table 1. A number of disease states were studied including cirrhosis (*n* = 5 studies) [6–10], pericarditis (*n* = 4 studies) [26, 27, 29, 31], gout (*n* = 5 studies) [15, 18, 34, 38, 39], knee osteoarthritis (*n* = 3 studies) [16, 20, 30], Behcet’s syndrome (*n* = 3 studies) [21, 32, 40], psoriatic arthritis (*n* = 2 studies) [13, 36], post-pericardiotomy syndrome (*n* = 2 studies) [25, 28], chronic obstructive pulmonary disorder (*n* = 1 study) [19], bare-metal stent restenosis (*n* = 1 study) [22], metabolic syndrome (*n* = 1 study) [23], lung resection surgery (*n* = 1 study) [17], myocardial infarction (n = 1 study) [37], familial Mediterranean fever (*n* = 1 study) [12], asthma (*n* = 1 study) [24], primary sclerosing cholangitis (*n* = 1 study) [11], aphthous stomatitis (*n* = 1 study) [33], allergic rhinitis (*n* = 1 study) [14] and low back pain (*n* = 1 study) [35]. Sample sizes ranged from 11 to 4745, with a pooled sample of 8659 adult participants. Mean age ranged from 27.0 to 69.1 years with most participants being male (73%). The inclusion and exclusion criteria reported by the included studies are shown in Supplementary Table 1.

**Table 1:** Characteristics of included studies

Reference	Country	Disease state	Intervention	Comparator	Daily dose of colchicine (mg)	Cumulative dose of colchicine (mg)	Length of intervention	Length of follow-up for AE assessment	Total sample size	Participant characteristics
Aran 2011 [16]	USA	Knee osteoarthritis	Colchicine, 0.5 mg twice daily	Placebo	1	91	3 months	3 months	61	Mean age 60.2 years 0% males
Batezzati 2001 [7]	Italy	Primary biliary cirrhosis	Colchicine, 1 mg daily + ursodeoxycholic acid, 250 mg daily	Placebo + ursodeoxycholic acid, 250 mg daily	1	3650	10 years	10 years	44	Mean age 56.5 years 14% males
Bessissow 2018 [17]	Canada	Lung resection surgery	Colchicine, 0.6 mg 3 times daily for first day, then twice daily	Placebo	1.26	12.6	10 days	30 days	100	Mean age 69 years 45% males
Borstad 2004 [18]	USA	Chronic gouty arthritis	Colchicine, 0.6 mg twice daily	Placebo	1.2	219.0	6 months	6 months	43	Mean age 63 years 86% males
Cohen 1991 [19]	USA	COPD in ex-cigarette smokers	Colchicine, 0.6 mg 3 times daily	Placebo	1.8	25.2	2 weeks	2 weeks	16	Mean age 65.9 years % males NR
Cortez-Pinto 2002 [8]	Portugal	Alcoholic cirrhosis	Colchicine, 1 mg daily, 5 days/week	Placebo	0.714	901.3	41.5 months (mean)	41.5 months (mean)	55	Mean age 53.8 years 89% males
Das 2002 [20]	India	Knee osteoarthritis	Colchicine, 0.5 mg twice daily + Piroxicam, 20 mg once daily	Placebo + Piroxicam, 20 mg once daily	1	152	5 months	5 months	39	Mean age 53 years 33% males
Davatchi 2009 [21]	Iran	Behcet’s disease	Colchicine, 1 mg daily	Placebo	1	122	4 months	4 months	282	Mean age 32.1 years 32% males
Deftereos 2013 [22]	Greece	Bare-metal stent restenosis in people with diabetes	Colchicine, 0.5 mg twice daily	Placebo	1	182	6 months	6 months	222	Mean age 63.6 years 65% males
Demidowich 2019 [23]	USA	Metabolic syndrome	Colchicine, 0.6 mg twice daily	Placebo	1.2	36.6	3 months	3 months	40	Mean age 45.8 years 23% males
Dinarello 1974 [12]	USA	Familial Mediterranean fever	Colchicine, 0.6 mg 3 times daily	Placebo	1.8	602.3	11 months	11 months	11	Mean age NR % males NR
Fish 1997 [24]	USA	Asthma	Colchicine, 0.6 mg twice daily	Placebo	1.2	50.4	6 weeks	6 weeks	71	Mean age 34 years 48% males
Imazio 2010 [25]	Italy	Post-periocardiotomy syndrome	Colchicine, 1 mg daily for first day then 0.5 mg daily for 1 month (< 70 kg) or 1 mg twice daily for first day then 0.5 mg twice daily for 1 month (≥70 kg)	Placebo	0.5 to 1	15 to 30	1 month	1 month	360	Mean age 66 years 66% males
Imazio 2011 [26]	Italy	Recurrent pericarditis	Colchicine, 1 mg daily for first day then 0.5 mg daily for 1 month (< 70 kg) or 1 mg twice daily for first day then 0.5 mg twice daily for 1 month (≥ 70 kg)	Placebo	0.5 to 1	91 to 182	6 months	18 months	120	Mean age 47.6 years 53% males
Imazio 2013 [27]	Italy	Acute pericarditis	Colchicine, 0.5 mg daily (< 70 kg) or 0.5 mg twice daily (≥ 70 kg)	Placebo	0.5 to 1.0	46 to 91	3 months	22 months (mean)	240	Mean age 52.1 years 60% males
Imazio 2014a [28]	Italy	Post-periocardiotomy syndrome and postoperative atrial fibrillation	Colchicine, 0.5 mg daily (< 70 kg) or 0.5 mg twice daily (≥ 70 kg)	Placebo	0.5 to 1.0	15 to 30	1 month	3 months	360	Mean age 67.5 years 69% males
Imazio 2014b [29]	Italy	Pericarditis	Colchicine, 0.5 mg daily (< 70 kg) or 0.5 mg twice daily (≥ 70 kg)	Placebo	0.5 to 1.0	91 to 182	6 months	18 months	240	Mean age 48.7 years 50% males
Kaplan 1986 [6]	USA	Primary biliary cirrhosis	Colchicine, 0.6 mg twice daily	Placebo	1.2	876.0	24 months	24 months	60	Mean age NR 5% males
Kershenobich 1979 [9]	Mexico	Cirrhosis	Colchicine, 1 mg daily, 5 days/week	Placebo	0.714	1042.4	48 months	48 months	43	Mean age 55.6 years 58% males
Kershenobich 1988 [10]	Mexico	Cirrhosis	Colchicine, 1 mg daily, 5 days/week	Placebo	0.714	1224.9	14 years (mean 4.7 years)	14 years (mean 4.7 years)	100	Mean age 50.3 years 49% males
Leung 2018 [30]	Singapore	Knee osteoarthritis	Colchicine, 0.5 mg twice daily	Placebo	1	112	16 weeks	16 weeks	109	Mean age 58.5 years 29% males
Liebenburg 2016 [31]	South Africa	Tuberculous pericarditis	Colchicine, 1 mg daily	Placebo	1	42	6 weeks	6 weeks	33	Mean age 31 years 33% males
Masuda 1989 [32]	Japan	Behcet’s disease	Colchicine, 1 mg daily	Cyclosporin, 10 mg/kg daily	1	112	16 weeks	16 weeks	96	Mean age NR % males NR
McKendry 1993 [13]	Canada	Psoriatic arthritis	Colchicine, 0.6 mg daily for first week, 0.6 mg twice daily for second week, 0.6 mg 3 times daily for 6 weeks	placebo	1.575	88.2	8 weeks	8 weeks	25	Mean age 40.7 years 56% males
Olsson 1995 [11]	Sweden	Primary sclerosing cholangitis	Colchicine, 1 mg daily	placebo	1	1095	36 months	36 months	84	Mean age 41.6 years 67% males
Pakfetrat 2010 [33]	Iran	Recurrent aphthous stomatitis	Colchicine, 0.5 mg daily	Prednisolone, 5 mg/d daily	0.5	47	3 months	6 months	34	Mean age 31.5 years 35% males
Paulus 1974 [34]	USA	Gout	Colchicine, 0.5 mg 3 times daily + probenecid 500 mg 3 times daily	Probenecid, 500 mg 3 times daily	1.5	274	6 months	6 months	52	Mean age 52.5 years 100% males
Roche 1995 [14]	France	Allergic rhinitis	Colchicine, 1 mg twice daily for 3 days then once daily for 5 days	Placebo	1.375	11	8 days	8 days	16	Age range 20 to 30 years 100% males
Schlesinger 2011 [15]	USA	Gout	Colchicine, 0.5 mg daily	Canakinumab dose ranging: single dose of 25 mg, 50 mg, 100 mg,200 mg, 300 mg or four doses at four-weekly intervals (50 mg, 50 mg, 25 mg, 25 mg)	0.5	56	16 weeks	24 weeks	432	Mean age 52.4 years 94% males
Schnebel 1988 [35]	USA	Low back pain	Colchicine, 0.6 mg hourly for 8 h then 0.6 mg every fourth day	Placebo	2.1	191.6	3 months	3 months	34	Mean age 69.1 years 63% males
Seideman 1987 [36]	Sweden	Psoriatic arthritis	Colchicine, 0.5 mg up to 3 times daily increased over 6 days	Placebo	0.5 to 1.5	30 to 91	2 months	2 months	15	Age range 20 to 65 years % males NR
Tardif 2019 [37]	Canada	Myocardial Infarction	Colchicine, 0.5 mg daily	Placebo	0.5	640.5	42 months	42 months	4745	Mean age 60.6 years 81% males
Terkeltaub 2010 [38]	USA	Gout	Colchicine, 1.2 mg followed by 0.6 mg in 1 h (low dose) or 1.2 mg followed by 0.6 mg hourly for 6 h (high dose)	Placebo	1.8 to 4.8	1.8 to 4.8	1–6 h	7 days	185	Mean age 51.5 years 95% males
Wang 2014 [39]	China	Gout	Colchicine, 0.5 mg twice daily for 3 days then once daily	Chuanhu anti-gout mixture, 250 ml daily	0.65	6.5	10 days	13.4 weeks	176	Mean age 52.8 years 94% males
Yurdakul 2001 [40]	Turkey	Behcet’s syndrome	Colchicine, 0.5 mg twice daily (≤50 kg), 0.5 mg alternating twice and 3 times daily (50–49 kg), 0.5 mg 3 times daily (60–75 kg), 0.5 mg alternating 3 and 3 times daily (76–84 kg), 0.5 mg 4 times daily (≥ 85 kg)	Placebo	1 to 2	730 to 1460	24 months	24 months	116	Mean age 27 years 52% males

*NR* Not reported

### Intervention characteristics

Of the 8659 pooled participants, 4225 participants were randomised to receive colchicine, 3956 to the placebo and 411 to an active comparator. The remaining 67 participants were included in cross-over trials and received both colchicine and placebo treatments over the duration of the study [12–15].

The length of treatment varied across studies (Table 1)). The majority of studies administered treatment for ≥ 1 to ≤ 6 months (*n* = 16 studies) [13, 15, 16, 20, 21, 23–25, 27, 28, 30–33, 35, 36], > 6 to ≤ 12 months (*n* = 6 studies) [12, 18, 22, 26, 29, 34] or > 12 to ≤ 48 months (*n* = 6 studies) [6, 8, 9, 11, 37, 40]. Four studies administered treatment for one to 2 weeks [14, 17, 19, 39] and participants in one study received treatment for one to 6 h [38]. Participants in two studies received treatment for ≥ 10 years [7, 10]. The mean daily dose of colchicine ranged from 0.5 mg to 4.8 mg. One study [38] reported the difference in the number of participants with adverse events based on whether they received low dose colchicine (total dose 1.8 mg) or high dose colchicine (total dose 4.8 mg).

### Adverse events

Methods used in the included studies to assess adverse events are described in Supplementary Table 1. Assessment methods included self-reporting of symptoms by patients, questioning of adverse events by investigators during study visits and undertaking blood tests and laboratory analyses.

#### Any adverse event

**Fig. 2:**
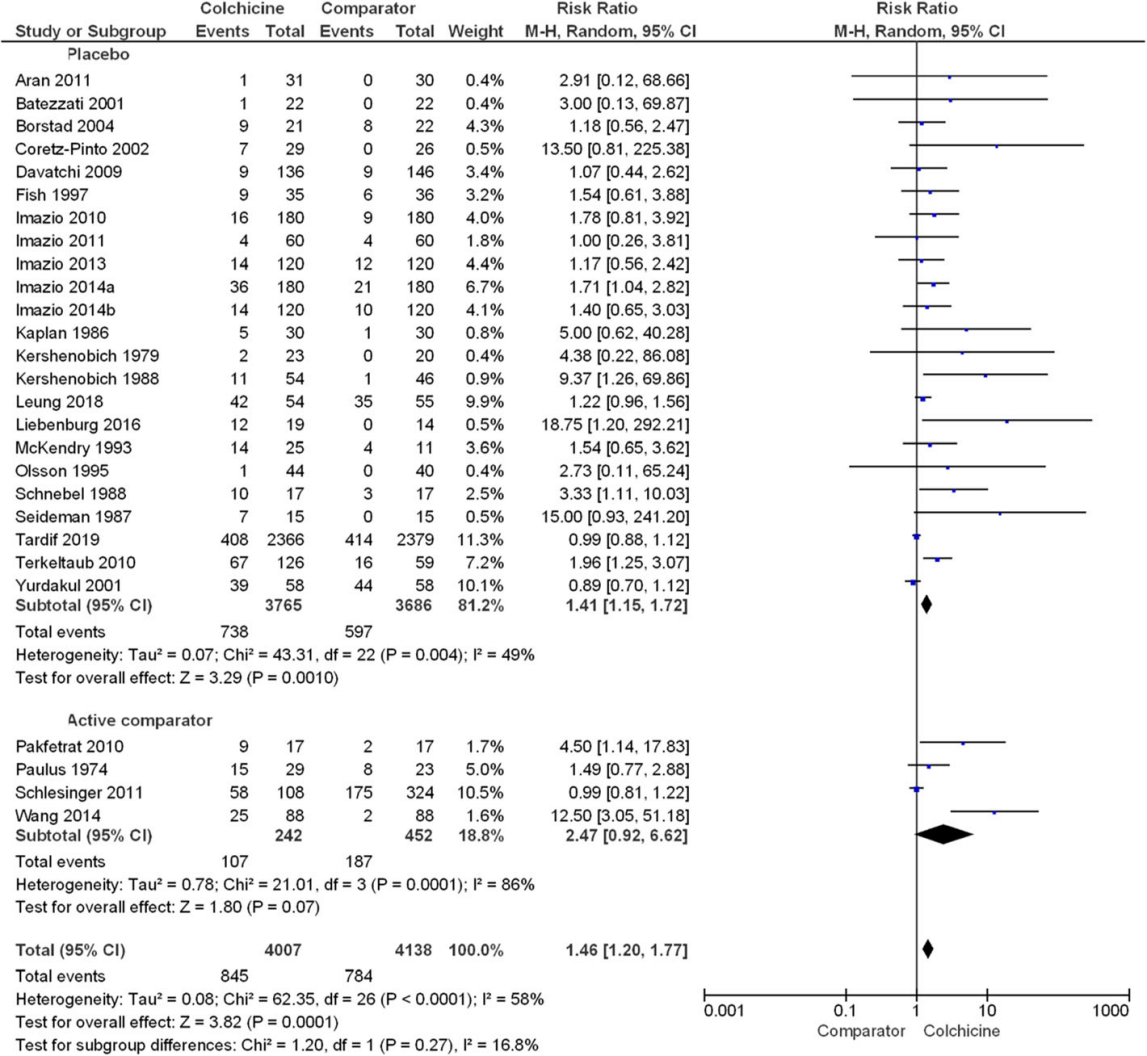
Forest plot showing estimated relative risk of any adverse event during colchicine use compared to placebo and active comparator groups

The number of participants with any adverse event was reported by 27 papers (Supplementary Table 2). From this data, 21.1% (95% confidence interval (CI) 19.9, 22.4) of participants using colchicine reported any adverse event compared to 18.9% (95% CI 17.7, 20.1) of participants in comparator groups. A meta-analysis showed the overall estimated risk ratio (RR) (95% CI) of any adverse event in colchicine users compared with pooled comparator groups was 1.46 (1.20, 1.77), *P* < 0.001 (Fig. 2, Table 2). The difference in RR of any adverse event in colchicine users was not significantly different between placebo and active comparator groups (*P* = 0.27). After the exclusion of six studies involving participants with liver disease, the RR (95% CI) of any adverse event in colchicine users vs. comparator groups was similar at 1.37 (1.14, 1.65), *P* < 0.001 (Supplementary Table 3).

**Table 2:** Meta-analysis results showing pooled risk ratio of adverse events between colchicine and pooled comparator groups

	*N*. studies	*n*/*N*, % (95% CI) participants		Pooled risk ratio (95% CI)	*I*^2^ (*P* value)	Overall effect, *Z* (*P* value)^a^
		Colchicine	Comparator			
Any event	27	845/4007, 21.1% (19.9, 22.4)	784/4152, 18.9% (17.7, 20.1)	1.46 (1.20, 1.77)	58% (< **0.001**)	3.82 (< **0.001**)
Diarrhoea	19	420/3212, 17.9% (16.8, 19.1)	262/3142, 13.1% (11.9, 14.3)	2.44 (1.62, 3.69)	58% (**< 0.001**)	4.24 (**< 0.001**)
Gastrointestinal^b^	29	729/4131, 17.6% (16.5, 18.8)	552/4213, 13.1% (12.1, 14.2)	1.74 (1.32, 2.30)	53% (**< 0.001**)	3.94 (**< 0.001**)
Liver	13	22/1150, 1.9% (1.2, 2.8)	15/1362, 1.1% (0.6, 1.8)	1.61 (0.86, 3.02)	0% (0.48)	1.50 (0.13)
Muscle^c^	9	37/872, 4.2% (3.0, 5.7)	29/869, 3.3% (2.3, 4.7)	1.25 (0.80, 1.93)	0% (0.69)	0.98 (0.33)
Haematology	8	16/2878, 0.6% (0.3, 0.9)	12/2893 0.4% (0.2, 0.7)	1.34 (0.64, 2.82)	0% (0.69)	0.77 (0.44)
Sensory^d^	2	3/201, 1.5% (0.4, 4.0)	2/190, 1.1% (0.2, 3.4)	1.35 (0.27, 6.74)	0% (0.58)	0.37 (0.71)
Infectious	7	105/2763, 3.8% (3.1, 4.6)	131/2997, 4.4% (3.7, 5.1)	1.03 (0.70, 1.51)	46% (0.09)	0.13 (0.90)

^a^Bolded *P* values indicate a significant overall effect in the risk ratio for an adverse event between colchicine and comparator groups

^b^The gastrointestinal category includes diarrhoea

^c^The muscle category includes myalgia, muscle cramps, myotoxicity, muscle weakness and elevated CPK. No rhabdomyolysis was assessed or reported by any study

^d^The sensory category includes dysthesia and paresthesia. No neuropathy was assessed or reported by any study

**Fig. 3:**
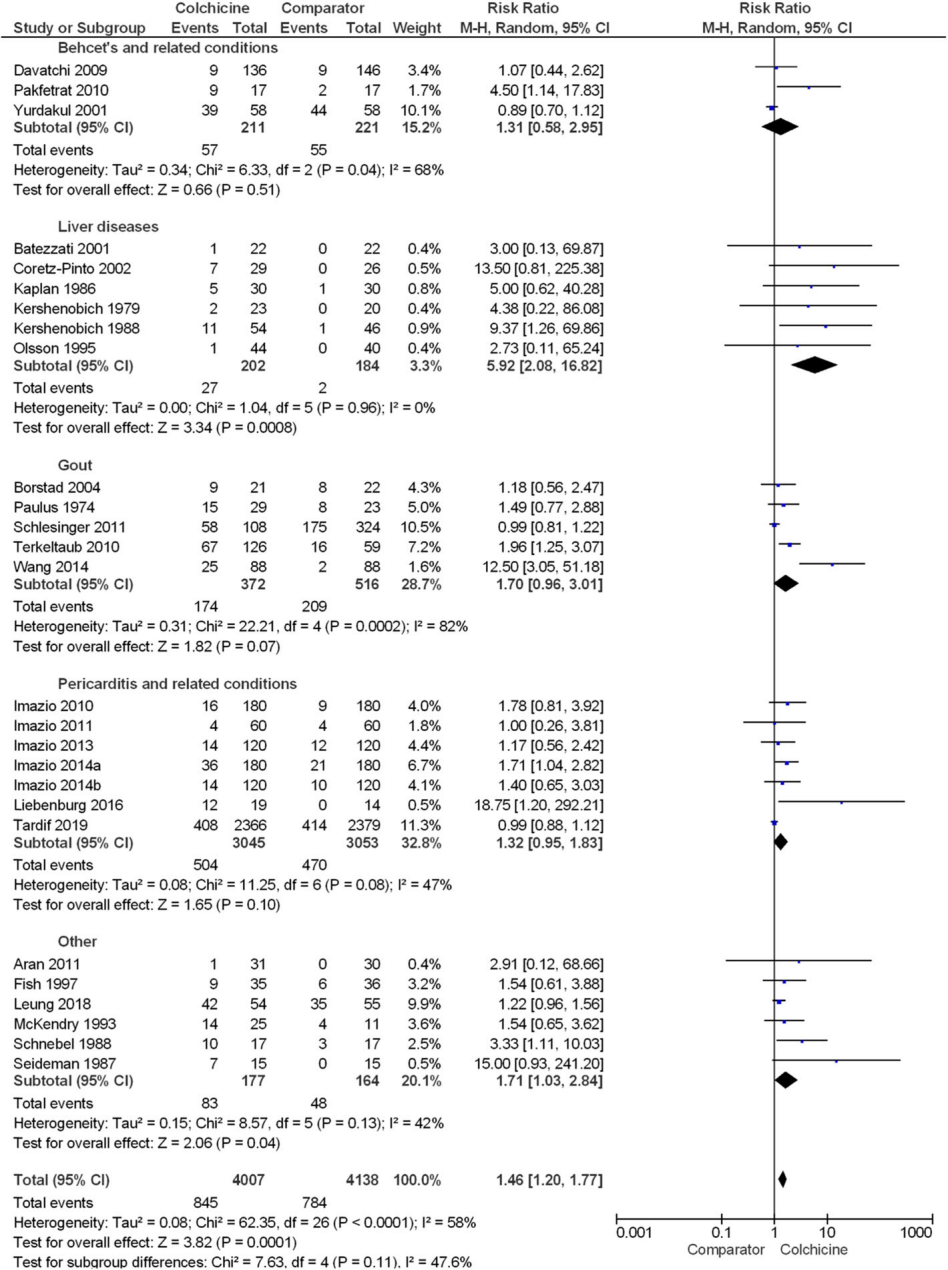
Forest plot showing estimated relative risk of any adverse event during colchicine use compared to comparator groups across different disease indications

**Fig. 4:**
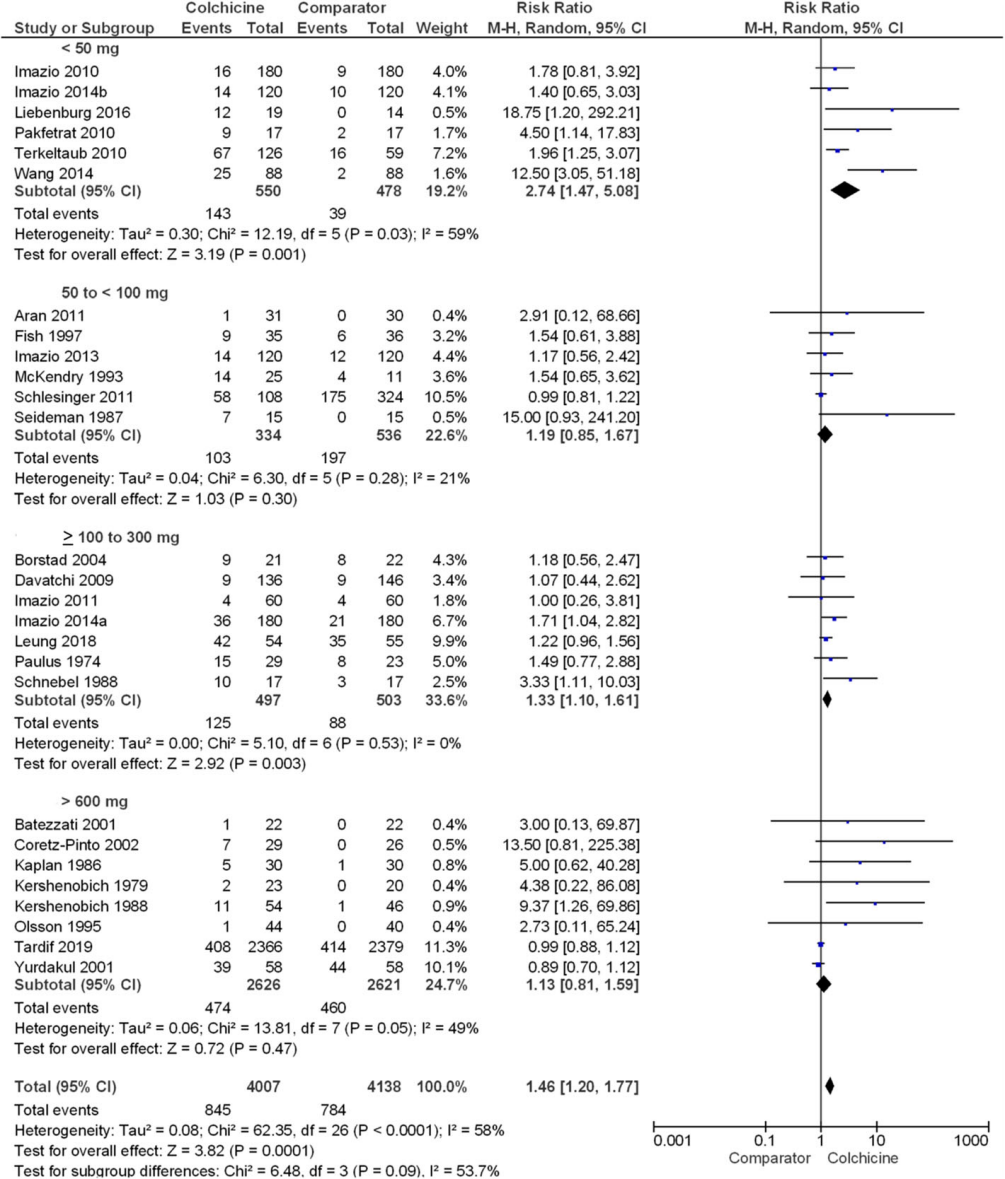
Forest plot showing estimated relative risk of any adverse event during colchicine use compared to comparator groups across different cumulative doses of colchicine

Although the sub-group meta-analyses showed a higher relative risk for any adverse event in colchicine users with liver diseases (RR 5.92 (95% CI 2.08, 16.82)), there was no overall significant difference in the relative risk of adverse events between different disease indications (*P* = 0.11) (Fig. 3). Furthermore, there was no significant difference in relative risk across different durations of drug exposure (*P* = 0.29) (Supplementary Figure 2), different colchicine daily dose categories (*P* = 0.70) (Supplementary Figure 3) or different colchicine cumulative dose categories (*P* = 0.09) (Fig. 4).

The proportion of participants with any adverse event computed from all 35 studies in this review (in which prevalence was considered 0% if not reported) was 20.6% (95% CI 19.5, 21.9) in colchicine users and 17.9% (95% CI 16.8, 19.1) in comparator groups.

#### Diarrhoea

The number of participants with diarrhoea was reported by a total of 19 papers (Supplementary Table 2). From this data, 17.9% (95% CI 16.8 19.1) of participants using colchicine reported diarrhoea compared to 13.1% (95% CI 11.9, 14.3) of participants in comparator groups. The meta-analysis showed the overall estimated RR (95% CI) of diarrhoea in colchicine users compared with pooled comparator groups was 2.44 (1.62, 3.69) (*P* < 0.001) ((Supplementary Figure 4, Table 2). The difference in RR between placebo and active comparator groups was not significant (*P* = 0.60). After exclusion of 6 studies involving participants with liver disease the RR (95% CI) of diarrhoea in colchicine users vs comparator groups was similar at 2.14 (1.40, 3.26), *P* < 0.001 (Supplementary Table 3).

The proportion of participants with diarrhoea computed from all 35 studies in this review (in which prevalence was considered 0% if not reported) was 10.8% (95% CI 9.9, 11.7) in colchicine users and 6.1% (95% CI 5.4, 6.8) in comparator groups.

#### Gastrointestinal adverse event

**Fig. 5:**
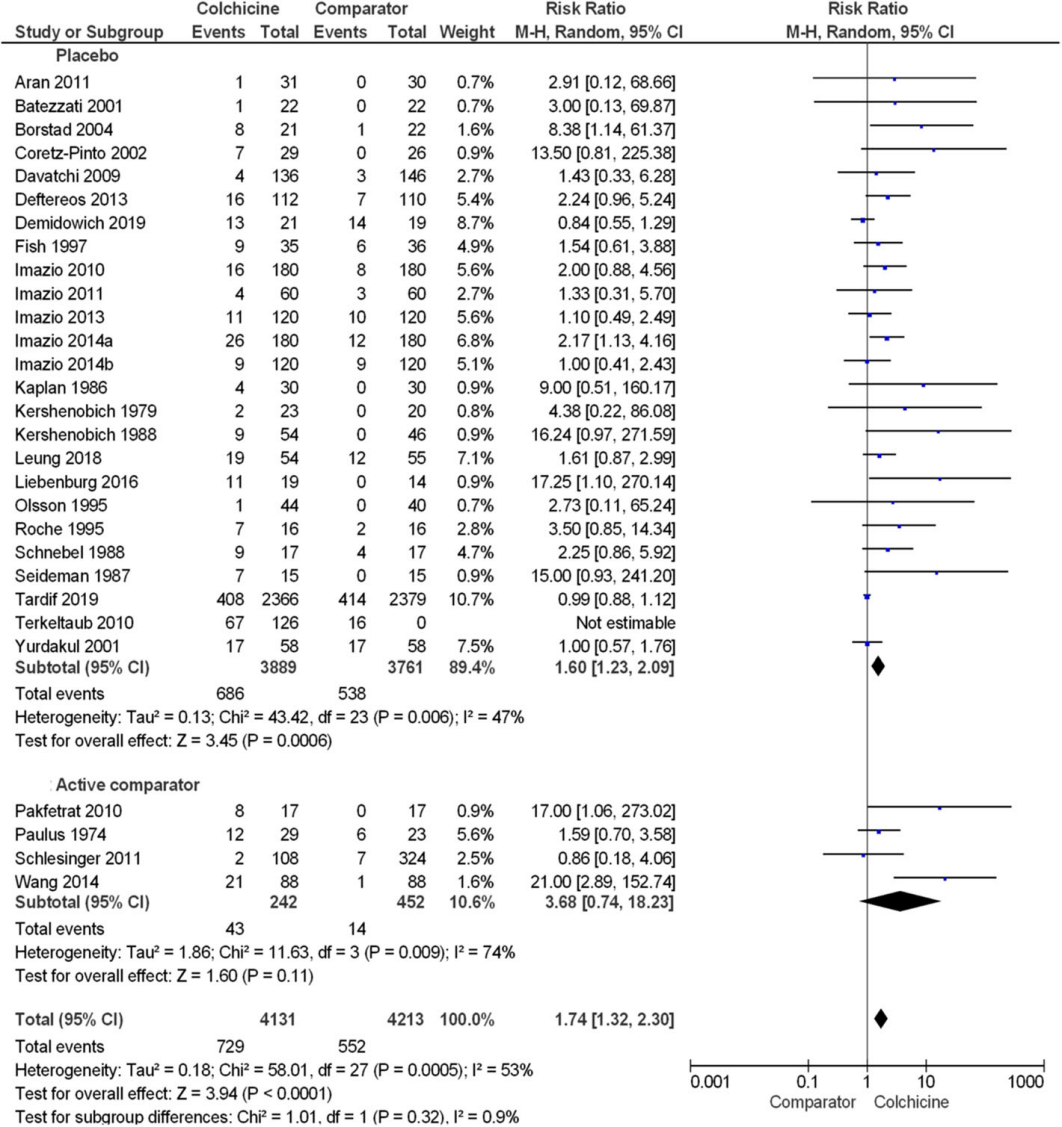
Forest plot showing estimated relative risk of any gastrointestinal event during colchicine use compared to placebo and active comparator groups

The number of participants with any gastrointestinal event was reported by 29 papers (Supplementary Table 2) and included diarrhoea, nausea, vomiting, abdominal pain, loss of appetite, bloating, constipation, melena and peptic ulcer (Supplementary Table 4). From these 29 papers, 17.6% (95% CI 16.5, 18.8) of participants using colchicine reported a gastrointestinal event compared to 13.1% (95% CI 12.1, 14.2) of participants in comparator groups. The overall RR (95% CI) of gastrointestinal events in colchicine users compared with pooled comparator groups was 1.74 (1.32, 2.30), *P* < 0.001 (Fig. 5, Table 2). The difference between placebo and active comparator groups was not significant (*P* = 0.32). After the exclusion of 6 studies involving participants with liver disease, the RR (95% CI) of any gastrointestinal event in colchicine users vs comparator groups was similar at 1.60 (1.22, 2.10), *P* < 0.001 (Supplementary Table 3).

The proportion of participants with any gastrointestinal event computed from all 35 studies in this review (in which prevalence was considered 0% if not reported) was 17.7% (95% CI 16.6, 18.8) in colchicine users and 12.6% (95% CI 11.6, 13.6) in comparator groups.

#### Liver events

The number of participants with liver events was reported by 13 papers (Supplementary Table 2) and included increased liver enzymes, hepatitis, hepatotoxicity and hepatic abnormalities (Supplementary Table 4). Pooled data from these papers showed 1.9% (95% CI 1.2, 2.8) of participants using colchicine reported a liver event compared to 1.1% (95% CI 0.6, 1.8) of participants in comparator groups. The overall RR (95% CI) of liver events in colchicine users did not significantly differ from the pooled comparator groups: 1.61 (0.86, 3.02) (Supplementary Figure 5, Table 2). The difference between placebo and active comparator groups was also not significant. None of the included papers involved participants with liver diseases.

The proportion of participants with any liver event computed from all 32 studies in this review (in which prevalence was considered 0% if not reported) was 0.5% (95% CI 0.3, 0.7) in colchicine users and 0.3% (95% CI 0.2, 0.5) in comparator groups.

#### Muscle events

**Fig. 6:**
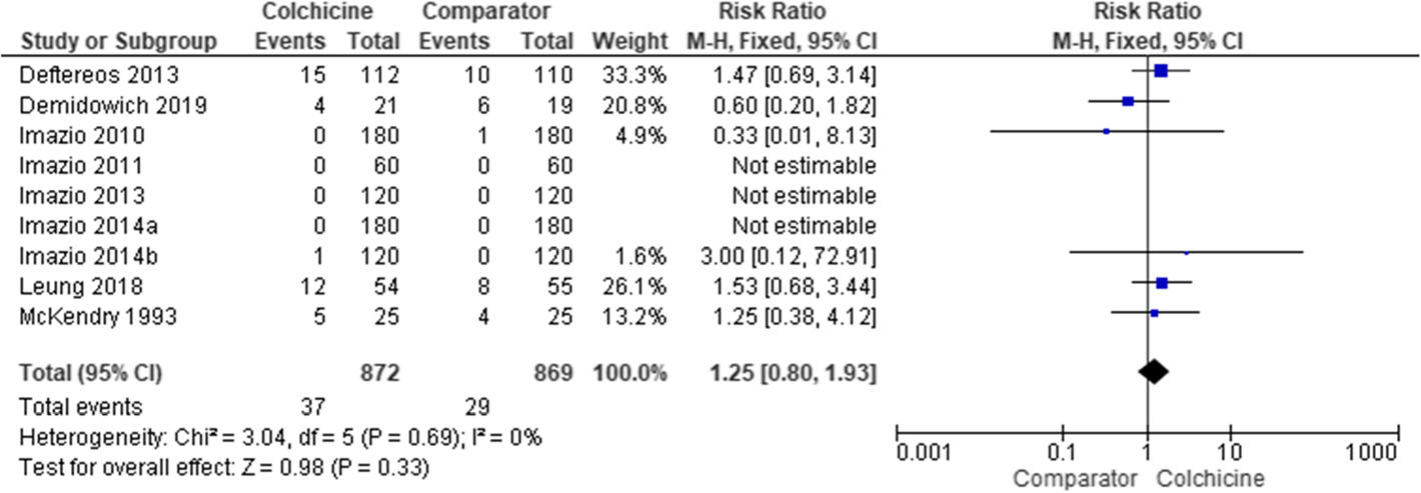
Forest plot showing estimated relative risk of muscle events during colchicine use compared to placebo (no active comparator studies)

The number of participants with muscle events was reported by nine studies (Supplementary Table 2) and included myalgia, muscle cramps, elevated creatine phosphokinase and muscle weakness (Supplementary Table 5). Rhabdomyolysis was not mentioned in any study. All nine studies involved placebo comparator groups. Pooled data from these studies showed 4.2% (95% CI 3.0, 5.7) of participants using colchicine reported a muscle event compared to 3.3% (95% CI 2.3, 4.7) of participants in placebo groups. The meta-analysis showed an overall non-significant RR (95% CI) of muscle events in colchicine users of 1.25 (0.80, 1.93) (Fig. 6, Table 2). None of the studies involved participants with liver diseases.

The proportion of participants with muscle events computed from all 35 studies in this review (in which prevalence was considered 0% if not reported) was 0.8% (95% CI 0.6, 1.1) in colchicine users and 0.6% (95% CI 0.4, 0.9) in comparator groups.

#### Haematology events

The number of participants with haematology events was reported by eight studies (Supplementary Table 2) and included anaemia, bone marrow toxicity, leukopenia and purpura (Supplementary Table 4). All studies involved placebo comparator groups. Pooled data from these studies showed 0.6% (95% CI 0.3, 0.9) of participants using colchicine reported a haematology event compared to 0.4% (95% CI 0.2, 0.7) of participants in placebo groups. The occurrence of haematology events in colchicine or comparator groups was reported by three studies [21, 23, 37]. The meta-analysis showed an overall non-significant RR (95% CI) of haematology events in 1.34 (0.64, 2.82) (Supplementary Figure 6, Table 2). None of the studies involved participants with liver diseases.

The proportion of participants with a haematology event computed from all 35 studies in this review (in which prevalence was considered 0% if not reported) was 0.4% (95% CI 0.2, 0.6) in colchicine users and 0.3% (95% CI 0.1, 0.4) in comparator groups.

#### Sensory events

No studies mentioned neuropathy-related adverse events. However, two studies involving placebo comparator groups reported other sensory events (Supplementary Table 2) which included dysthesia in the legs and paresthesia (Supplementary Table 5). From this data, the pooled prevalence of sensory events was 1.1% (95% CI 0.2, 3.4) in colchicine users and 1.5% (95% CI 0.4, 4.0) in placebo groups. The meta-analysis showed an overall non-significant RR (95% CI) of sensory events in colchicine users of 1.35 (0.27, 6.74) (Supplementary Figure 7, Table 2). None of the included papers involved participants with liver diseases.

The proportion of participants with any sensory events computed from all 35 studies in this review (in which prevalence was considered 0% if not reported) was 0.04% (95% CI 0.0, 0.1) in colchicine users and 0.07% (95% CI 0.0, 0.2) in comparator groups.

#### Infectious events

Seven studies reported various infectious events (Supplementary Table 2), including urinary tract infection, parotiditis, shingles, upper respiratory tract infection, nasopharyngitis and sinus congestion (Supplementary Table 5). From these papers, 0.4% (95% CI 0.2, 0.6) of participants using colchicine reported an infectious event compared to 2.1% (95% CI 1.6, 2.7) of participants in comparator groups. The overall RR (95% CI) of infectious events in colchicine users compared with pooled comparator groups was non-significant: 1.03 (0.70, 1.51) (Supplementary Figure 8, Table 2). The difference between placebo and active comparator groups was not significant (*P* = 0.94). No study involved participants with liver diseases.

The proportion of participants with any infectious event computed from all 35 studies in this review (in which prevalence was considered 0% if not reported) was 2.4% (95% CI 2.0, 2.9) in colchicine users and 2.8% (95% CI 2.4, 3.4) in comparator groups.

#### Death

Death related to adverse events was specifically reported in three studies (Supplementary Table 2). No study reported deaths related to an adverse event.

#### Miscellaneous events

Miscellaneous adverse events reported by the included studies are summarised in Supplementary Table 6. These events were not meta-analysed but contributed to the ‘any adverse event’ category.

## Discussion

This systematic review and meta-analysis of randomised controlled trials indicate that overall, colchicine increases the rate of adverse events compared to both placebo and active comparators. Analysis of individual events demonstrated an increased risk for diarrhoea and gastrointestinal events in colchicine users, but no increase in the rate of other commonly cited adverse events, including liver, muscle, haematology, sensory or infectious events.

The mechanism by which colchicine induces diarrhoea and other gastrointestinal symptoms is not exactly known, but can be attributed to an increase in prostaglandin synthesis, intestinal secretion and gastrointestinal motility with this drug [41]. Although these symptoms can be clinical features of colchicine toxicity, they are usually mild, short-lived and reversible with dose reduction [12]. Serious adverse events associated with colchicine use, including neuropathy, myotoxicity and death were not reported in any trial included in the current analysis. These events may be more readily observed in less controlled environments evident in case reports involving colchicine over-dose, chronic renal diseases, interaction with concomitant medications and intravenous administration [42–49].

Analysis of adverse events in colchicine users showed no difference across different disease indications. Although overall, adverse events were numerically higher in patients with liver diseases, this risk was not significantly different from other disease indications. Furthermore, the sub-analysis excluding participants with liver disease showed similar adverse events rates to the main analysis. Although dose reduction is generally recommended when colchicine is used continuously in those with severe renal impairment, accurate conclusions regarding adverse events in this population could not be drawn from the current analysis.

There was notable heterogeneity across the clinical trials included in this review with regards to intervention methodology, including colchicine dose and treatment duration. However, sub-group analyses concluded that differences in drug use duration, daily dose or cumulative dose categories had no effect on the risk for adverse events. This contrasts with trials assessing the treatment of acute gout which report that high-dose colchicine results in a greater risk-to-benefit ratio. The paper reporting the AGREE trial included in the current analysis by Terkeltaub et al. [38], which directly compared two different doses of colchicine, found differences in adverse event rates between low and high dose groups, with 36% and 81% of participants having any adverse event, respectively. However, the short duration of treatment (1 to 6 h) meant that the cumulative drug doses in both groups were low in the context of the other papers included in the meta-analysis, resulting in a non-significant effect of dose in the meta-analysis. The difference in adverse event rates between the two arms of this AGREE trial may relate to better surveillance of adverse events in this trial compared to previously reported trials, or the relatively high dose (4.8 mg over 6 h) of the high-dose colchicine group. This is the only published trial comparing two differing doses of colchicine so conclusions on the reason for this disparity are difficult to be definitive about.

The limitations of this study include the inability in assessing the occurrence of rarer adverse events when only short duration controlled clinical trials were included. Different methodology is required to assess the frequency of rarer adverse events. Furthermore, the aims of the majority of the included studies were not primarily to assess safety, resulting in limited availability of adverse event data for extraction. As only studies which specifically reported an adverse event as being present or absent were included in the meta-analyses, it is possible that the pooled results may have over-estimated the true occurrence of adverse events which were not reported in all papers. In addition, it is also possible that the pooled results may have under-estimated the true occurrence of adverse events which were not assessed (e.g. those requiring blood tests). There were few included participants with severely impaired renal function, so the ability to assess for safety in this group was limited. Clinical trials often recruit patients in a highly selective manner, including excluding those with co-morbidities, and therefore the results are not necessarily generalizable to a general patient population. In addition, the included studies spanned over 20 years and it is likely that participants in earlier studies are not representative of patients treated with colchicine in clinical practice today. Other limitations include the screening of titles, abstracts and full-texts being undertaken by a single reviewer, and the exclusion of non-English language publications.

The strengths of this study include the strict inclusion of only placebo or active comparator blinded trials which reduces the potential for bias; although the occurrence of diarrhoea in participants can lead to at the least the suspicion of being in the colchicine group. In addition, there was a wide range of included indications such as gout, familial Mediterranean fever, Behcet’s disease and pericarditis, which leads to increased generalisability of the study results.

## Conclusions

This meta-analysis provides reassurance that common adverse events with colchicine use are limited to diarrhoea and gastrointestinal events. Whilst these are not benign side effects in some individuals, they will settle on dose reduction or drug discontinuation. More serious adverse events during colchicine use, including liver and haematological changes, muscle toxicity, neuropathy and death are very infrequent in clinical trials.

## Supplementary information

**Supplementary Table 1.** Participant inclusion and exclusion criteria and adverse event assessment methods of included studies. Table 2. Frequency of any adverse event reported in colchicine and comparator groups. Table 3. Meta-analysis results showing pooled risk ratio of adverse events between colchicine and pooled comparator groups for studies not involving participants with liver diseases. Table 4. Number of participants in colchicine and comparator groups with adverse events related to gastrointestinal, liver and hematologic events. Table 5. Number of participants in colchicine and comparator groups with adverse events related to muscle, sensory, and infectious events. Table 6. Number of participants in colchicine and comparator groups with miscellaneous adverse events or death. Figure 1. Quality assessment results using the modified-Jadad score. Figure 2. Forest plot showing estimated relative risk of any adverse event during colchicine use compared to comparator groups across different durations of drug exposure. Figure 3. Forest plot showing estimated relative risk of any adverse event during colchicine use compared to comparator groups across different daily doses of colchicine. Figure 4. Forest plot showing estimated relative risk of diarrhoea during colchicine use compared to placebo and active comparator groups. Figure 5. Forest plot showing estimated relative risk of liver events during colchicine use compared to placebo and active comparator groups. Figure 6. Forest plot showing estimated relative risk of hematology events during colchicine use compared to placebo (no active comparator studies). Figure 7. Forest plot showing estimated relative risk of sensory events during colchicine use compared to placebo (no active comparator studies). Figure 8. Forest plot showing estimated relative risk of infectious events during colchicine use compared to placebo and active comparator groups.

## Data Availability

The data is available on request to the corresponding author.

## Abbreviations

CI: Confidence interval
PRISMA: Preferred Reporting Items for Systematic Reviews and Meta-Analyses
RR: Risk ratio
