# Supplementary material for: Adverse events during oral colchicine use: a systematic review and meta-analysis of randomised controlled trials
Source: Arthritis Res Ther. 2020 Feb 13;22:28. doi: 10.1186/s13075-020-2120-7 (PMC7020579; doi:10.1186/s13075-020-2120-7)
Supplement: Supplementary file 1 — Supplementary Table 1. Participant inclusion and exclusion criteria and adverse event assessment methods of included studies. Table 2. Frequency of any adverse event reported in colchicine and comparator groups. Table 3. Meta-analysis results showing pooled risk ratio of adverse events between colchicine and pooled comparator groups for studies not involving participants with liver diseases. Table 4. Number of participants in colchicine and comparator groups with adverse events related to gastrointestinal, liver and hematologic events. Table 5. Number of participants in colchicine and comparator groups with adverse events related to muscle, sensory, and infectious events. Table 6. Number of participants in colchicine and comparator groups with miscellaneous adverse events or death. Figure 1. Quality assessment results using the modified-Jadad score. Figure 2. Forest plot showing estimated relative risk of any adverse event during colchicine use compared to comparator groups across different durations of drug exposure. Figure 3. Forest plot showing estimated relative risk of any adverse event during colchicine use compared to comparator groups across different daily doses of colchicine. Figure 4. Forest plot showing estimated relative risk of diarrhoea during colchicine use compared to placebo and active comparator groups. Figure 5. Forest plot showing estimated relative risk of liver events during colchicine use compared to placebo and active comparator groups. Figure 6. Forest plot showing estimated relative risk of hematology events during colchicine use compared to placebo (no active comparator studies). Figure 7. Forest plot showing estimated relative risk of sensory events during colchicine use compared to placebo (no active comparator studies). Figure 8. Forest plot showing estimated relative risk of infectious events during colchicine use compared to placebo and active comparator groups. [file 13075_2020_2120_MOESM1_ESM.docx]

| **Supplementary Table 1.** Participant inclusion and exclusion criteria and adverse event assessment methods of included studies | | | |
| --- | --- | --- | --- |
|  | **Inclusion criteria** | **Exclusion criteria** | **Adverse event assessment method** |
| Aran 2011 [[24](#_ENREF_24)] | -Postmenopausal women.  -Primary diagnosis of moderate-to-severe knee osteoarthritis according to the ACR criteria and the Oxford Knee Score. | -Clinical or radiographic evidence of RA or other immunologic diseases.  -GI upset.  -Renal or hepatic disease.  -History of allergy.  -Intra-articular injections in past 3 months.  -Contraindications to the use of colchicine. | -Assessment method of AEs not specified. |
| Batezzati 2001 [[6](#_ENREF_6)] | -Established diagnosis of primary biliary cirrhosis. | -Severe liver disease (defined by ascites, previous episodes of gastrointestinal bleeding or encephalopathy, serum bilirubin levels exceeding 10 mg/dL).  -Evidence of malignant conditions or of other major diseases unrelated to primary biliary cirrhosis.  -Alcohol abuse  -Previous treatment with colchicine or immunosuppressant agents.  -If low compliance was anticipated. | -Assessment method of AEs not specified. |
| Bessissow 2018 [[36](#_ENREF_36)] | -Aged > 55 years.  -In sinus rhythm undergoing a tumour resection in the lung (malignant, benign or unknown). | -In atrial fibrillation or flutter just prior to surgery.  -Contraindications to colchicine (i.e., allergy, myelodysplastic disorders, pregnancy, or e-GFR <30 ml/min/1.73m2.  -Patient not expected to take oral medications for >24 hours after surgery (e.g., esophagectomy).  -Prior use of colchicine. | -Assessment method of AEs not specified. |
| Borstad 2004 [[20](#_ENREF_20)] | -Aged > 19 years.  -Presence of tophi.  -Uric acid overproduction.  -Frequent attacks of gout (≥ 3 attacks/year).  -Elevated serum urate in the setting of chronic renal insufficiency.  -Nephrolithiasis. | -Use of chronic colchicine within the past 3 months.  -History of allergic reaction to allopurinol or colchicine.  -Severe renal insufficiency (creatinine clearance < 20 ml/min).  -Female with childbearing potential.  -Evidence of active hepatitis. | -Diarrhea was self-reported.  -Assessment method of other AEs not specified. |
| Cohen 1991 [[33](#_ENREF_33)] | -Ex-cigarette smoker.  -Irreversible airflow obstruction.  -Aged between 45-75 years. | -Other medical illnesses such as heart failure or diabetes.  -Using steroids, antibiotics, or NSAIDs. | -Liver function tests and complete blood counts were measured in venous blood before and after treatment. |
| Cortez-Pinto 2002 [[7](#_ENREF_7)] | -Ambulatory.  Aged 18-65 years.  -Biopsy-proven liver cirrhosis.  -A well-documented history of previous daily alcohol intake for more than 5 years. | -Presence of other liver diseases, namely haemochromatosis, Wilson's disease, a1-antitrypsin deficiency, autoimmune hepatitis, primary biliary cirrhosis, or viral hepatitis.  -Child-Pugh class C.  -Serum bilirubin >10 mg/dl.  -Gastrointestinal bleeding in the previous 15 days  -Refractory ascites.  -Serious illness, e.g. renal failure, cardiac failure or neoplasia. | -Evaluated at each study visit through questioning patients. |
| Das 2002 [[25](#_ENREF_25)] | -Aged 40-75 years.  -Fulfilled the ACR criteria for knee osteoarthritis. | -Clinical or radiological evidence of rheumatoid arthritis or other immunological diseases.  -Renal/hepatic diseases.  -Allergies.  -Contraindications to the use of piroxicam, colchicine and intraarticular steroids. | -AEs were recorded in an open format for new problems and a visual analogue scale for abdominal problems. |
| Davatchi 2009 [[27](#_ENREF_27)] | -Aged between 14-60 years.  -Confirmed diagnosis of Behcet’s disease.  -At least one active symptom.  -No treatment for at least 1 month. | -Major organ involvement (eye, brain, major intestinal, lung, and cardiovascular involvement) | -Liver function assessed by measuring liver enzymes  -Urinary tract infection self-reported  -Assessment method of other AEs not specified |
| Deftereos 2013 [[34](#_ENREF_34)] | -Diagnosed diabetes mellitus.  -Treated with oral medication or insulin.  -Aged 40-80 years.  -Undergoing percutaneous coronary intervention with bare metal stent. | -Left main artery disease  -Coronary intervention performed as primary treatment for ST-segment elevation myocardial infarction.  -Hepatic impairment.  -Target vessel segment presenting particular technical challenges for intravascular ultrasound.  -Severe or end-stage renal failure (e-GFR 20 ml/min/1.73 m2 or requiring dialysis).  -History of intolerance to colchicine.  -Myopathy, statin hepatotoxicity or myotoxicity. -Women with child-bearing potential. | -AEs were monitored at clinical visits and through complete blood counts and standard biomechanical analyses performed at regular intervals. |
| Demidowich 2019 [[35](#_ENREF_35)] | -Aged ≥ 18 years.  -Obesity (BMI ≥ 30 kg/m^2^).  -Evidence of inflammation (high-sensitivity CRP ≥ 2.0 mg/L).  -Insulin resistance.  -American Heart Association diagnosis of Metabolic syndrome. | -Significant medical illness (e.g. diabetes mellitus, uncontrolled hypertension, congestive heart failure).  -eGFR <60 mL/min/1.73 m2. -Recent/current tobacco, nicotine, or illicit substance use.  -Previous history of agranulocytosis, significant myositis, or gout.  -Recent/current use of colchicine or medication known to affect colchicine metabolism/clearance.  -Known allergy to colchicine  -Recent or current use of other anti-inflammatory medications (e.g. aspirin, other NSAIDs, corticosteroids)  -Recent/current use of medication known to affect glucose or body weight  -Change in body weight >3% in the 2 months prior to enrolment.  -For women: irregular menses, pregnancy, breastfeeding, or planning pregnancy in the next 6 months, unwilling to use contraception during the study. | - AEs were assessed through clinician interviews using a structured  questionnaire containing a list of symptoms designed to identify  potential adverse drug reactions (i.e. self-reported by patients). Adverse events were graded  according to the Common Terminology Criteria for Adverse Events (CTCAE).  -Blood tests were also undertaken at baseline and follow-up to assess hematological events. |
| Dinarello 1974 [[12](#_ENREF_12)] | -History of frequent attacks of familial Mediterranean fever.  -Were familiar with characteristics of their disease. | -Any hepatic, renal, and hematologic abnormalities measured by usual laboratory criteria.  -A rectal biopsy positive for amyloid. | -Assessment method of AEs not specified |
| Fish 1997 [[38](#_ENREF_38)] | -Male and non-pregnant female subjects  -Aged 18-60 years  -Moderate asthma  -Used inhaled corticosteroids for control of symptoms for at least 30 days prior to entry.  -Treatment failure asthma. | -NA | -Assessment method of AEs not specified |
| Imazio 2010 [[31](#_ENREF_31)] | -Candidate for cardiac surgery.  -Aged ≥18 years | -Contraindication to colchicine.  -Unfavourable short-term outlook.  -Known severe liver disease or current transaminases >1.5 times the upper normal limit.  -Current serum creatinine above 2.5 mg/dL.  -Known myopathy or elevated baseline pre-operative creatine kinase.  -Known blood dyscrasias or gastrointestinal disease.  -Pregnant and lactating women or women of childbearing potential not protected by a contraception method.  -Known hypersensitivity to colchicine, or current treatment with colchicine for any indications. | - Monitoring and recording of all AEs was performed during follow-up and monitored by a safety committee.  - Blood tests for liver, muscle and bone marrow toxicity. |
| Imazio 2011[[16](#_ENREF_16)] | -Definite diagnosis of recurrent pericarditis.  -Aged 18 years or older.  -Had favourable short-term prognosis. | -First episode of acute pericarditis or their second or subsequent recurrence.  -Had pericarditis with tuberculous, purulent, or neoplastic causes.  -Known severe liver disease.  -Current aminotransferase levels greater than 1.5 times the upper limit of normal.  -Current serum creatinine level greater than 221 mol/L.  -Known myopathy.  -Serum creatine kinase level above the upper limit of normal.  -Known blood dyscrasias.  -Gastrointestinal disease  -Known hypersensitivity to colchicine.  -Pregnant or lactating women and women in their childbearing years who were not using contraception.  -Receiving or had previously received colchicine for any indication. | -AEs were monitored by a safety committee and recorded during follow-up and filed according to whether it was patient-reported or determined through blood chemistry. |
| Imazio 2013 [[17](#_ENREF_17)] | -Aged 18 years or older.  -First episode of acute pericarditis. | -Tuberculous, neoplastic, or purulent pericarditis.  -Severe liver disease or current aminotransferase levels of more than 1.5 times the upper limit of the normal range.  -A serum creatinine level of more than 2.5 mg/dL.  -Skeletal myopathy or a serum creatine kinase level above the upper limit of the normal range.  -Blood dyscrasia.  -Inflammatory bowel disease.  -Hypersensitivity to colchicine or other contraindication to its use.  -Current treatment with colchicine.  -Life expectancy of 18 months or less.  -Pregnant or lactating women or women of childbearing potential who were not protected by a contraception method.  -Evidence of myopericarditis, as indicated by an elevation in the serum troponin level. | -AEs were monitored and recorded during follow-up by a safety committee.  -Blood chemical analyses were performed at each study visit to assess for liver and muscle toxicity. |
| Imazio 2014a [[32](#_ENREF_32)] | -Patients undergoing cardiac surgery for any reason excluding cardiac transplantation. | -Absence of sinus rhythm at enrolment. -Cardiac transplantation.  -Contraindications to colchicine. | -AEs were monitored by a safety committee and recorded during follow-up and filed according to whether it was patient-reported or determined through blood chemistry. |
| Imazio 2014b [[18](#_ENREF_18)] | -Aged 18 years or older.  -Two or more recurrences of pericarditis (idiopathic, viral, post-cardiac injury, or caused by connective tissue disease). | -Tuberculous, neoplastic, or purulent pericarditis  -Severe liver disease or current aminotransferase concentrations more than 1.5 times the upper limit of the normal.  -Serum creatinine concentration more than 221.00 μmol/L.  -Skeletal myopathy or serum creatine kinase concentration more than the upper limit of the normal.  -Blood dyscrasia.  -Inflammatory bowel disease.  -Hypersensitivity to colchicine or other contraindication to colchicine.  -Current treatment with colchicine.  -Life expectancy of 18 months or less. -Pregnant or lactating women or women of childbearing potential not using contraception.  -Evidence of myopericarditis as indicated by any increase of serum troponin concentration. | -During follow-up, all adverse events were monitored and recorded.  -Blood chemistry analyses were undertaken at each visit to test for muscle and liver toxicity. |
| Kaplan 1986 [[8](#_ENREF_8)] | -Clinical history and biochemical profile consistent with primary biliary cirrhosis.  -A positive test for antimitochondrial antibody.  -Liver-biopsy results consistent with or diagnostic of primary biliary cirrhosis.  -Radiologic or ultrasonographic evidence that the bile ducts were patent. | -Concomitant debilitating cardiovascular disease.  -Advanced end-stage liver disease. | -Assessment method of AEs not specified |
| Kershenobich 1979 [[9](#_ENREF_9)] | -Histologic evidence of liver cirrhosis.  -Prothrombin time below 17 seconds.  -Serum total bilirubin below 2 mg. | -Evidence of chronic active hepatitis with liver cirrhosis, and episodes of gastrointestinal bleeding.  -Encephalopathy during a period of 2 weeks before the trial.  -Inability to attend the clinic regularly for geographical or other reasons. | -Assessment method of AEs not specified |
| Kershenobich 1988 [[10](#_ENREF_10)] | -A definitive diagnosis of liver cirrhosis.  -Aged 18 years or older. | -An episode of gastrointestinal bleeding or encephalopathy within two weeks before entry into the trial.  -A total serum bilirubin level below 171 umol/L or serum albumin level below 220 umol/L.  -Severe concomitant disease.  -Unable to attend the clinic regularly for geographic or other reasons. | -AEs were recorded at clinical evaluations with patients.  -Blood chemistry tests were used to measure serum levels for muscle toxicity and hematologic abnormalities.  -Liver screening was performed every 6 to 12 months. |
| Leung 2018 [[26](#_ENREF_26)] | -Symptomatic knee osteoarthritis diagnosed according to ACR criteria.  -Kellgren Lawrence grade 2 in at least one knee on radiography.  -Responded positively to “Do you have pain, aching or stiffness of the knee on most days of the past month?”.  -Knee pain score of 40/100 on a visual analogue scale. | -Other joint diseases, inflammatory arthritis, history of gout or podagra.  -Knee arthroscopy within 6 months.  -Joint replacement in either knee or anticipating knee replacement in next 4 months.  -Significant renal, liver or muscle impairment, pregnancy or cognitive impairment.  -On treatment with drugs known to inhibit cytochrome 450 (CYP3A4) and/or P-glycoprotein15, which increases the risk of colchicine-induced toxicity. | -AEs were assessed at each visit using open-ended questions and a checklist for diarrhea, myalgia and weakness, and physical examination of muscle tenderness and strength.  -Laboratory assessments included renal and liver function tests and creatinine phosphokinase (CPK). |
| Liebenburg 2016[[19](#_ENREF_19)] | -Patients with definite or probable tuberculous pericarditis. | -Renal or hepatic impairment (creatinine clearance rate < 85 ml/min or transaminases > 1.5 upper limit of normal).  -Pregnant patients or patients intending to become pregnant within four months. | -Assessment method of AEs not specified. |
| Masuda 1989 [[28](#_ENREF_28)] | -Aged 15 years or more.  -Complete or incomplete Behcet’s disease.  -Visual acuity of 20/40 or less.  -Experienced at least two episodes of ocular attacks during the 16 weeks before the study. | -Renal or hepatic dysfunction  -Neurological Behcet’s disease  -Hypertension | -Assessment method of AEs not specified. |
| McKendry 1993 [[13](#_ENREF_13)] | -Aged 18 years or older.  -Psoriasis diagnosed by a dermatologist.  -Active synovitis (tenderness or effusion, or both) in three or more joints. | -Women with inadequate contraception.  -History of inflammatory bowel disease.  -Liver disease.  -Renal disease.  -Bone marrow hypoplasia.  -Increased creatine kinase.  -Rheumatoid factor (latex) test greater than 1/80. | -AEs were assessed at each visit by questioning patients for possible adverse effects or drug changes. |
| Olsson 1995 [[11](#_ENREF_11)] | -Diagnosis of primary sclerosing cholangitis. | -Not specified. | -Blood chemistry analysis was performed at least 6 monthly to test for hematologic side effects and myopathy.  -Patients recorded daily on prefabricated 1-month charts, the occurrence of pruritus, fever, "biliary  pain," and other possible symptoms on a scale of 0-3 (0, no symptom; 1, symptom that can easily be disregarded; 2, symptom that is disturbing but leads only to occasional interruption of work; and 3, symptom that causes the patient to stop work  or disturbs sleep at night). |
| Pakfetrat 2010 [[39](#_ENREF_39)] | -Patients with a history of recurrent apthoous stomatitis (at least three episodes each month).  -Unresponsive to conventional topical treatments.  -Not taking treatment for stomatitis in the two weeks prior to the beginning of the study. | -Previous medical history of any systemic disease (including diabetes mellitus, blood dyscrasia, liver disease, inflammatory bowel disease, renal insufficiency and rheumatologic diseases such as Behcet's disease).  -Had taken any medicine that might have an effect on the immune system (such as glucocorticoids).  -Had involvement of other mucous membranes. | -The patients were asked to report immediately if there was any adverse event at any time of study until six months after treatment.  -Patients were also assessed for any possible adverse events by researchers at each appointment every 2 weeks. |
| Paulus 1974 [[21](#_ENREF_21)] | -Male.  -Confirmed gout. | -Known to be uncooperative during treatment.  -Significant renal disease (serum creatinine > 1.2 mg/100 ml). | -Side effects were reported during un-suggestive questioning at each monthly visit. |
| Roche 1995 [[14](#_ENREF_14)] | -Non-smoking.  -Male patients.  -Confirmed allergic rhinitis.  -Normal clinical examination, anterior rhinoscopy, electrocardiogram and routine biologic tests | -History of respiratory infection during the 6 weeks preceding study.  -Any chronic bronchopulmonary disease.  -Took medications during the previous 2 weeks or during study. | -Assessment method of AEs not specified. |
| Schlesinger 2011[[15](#_ENREF_15)] | -Aged 18–80 years.  -Diagnosis of gouty arthritis as defined by the ACR criteria.  -At least two gouty arthritis flares in the previous year.  -Body mass index of ≤40 kg/m. | -Had a gouty arthritis flare within 2 weeks of screening.  -History of allergy.  -Contraindication, or intolerance to allopurinol, colchicine, naproxen or oral prednisolone/prednisone.  -History of bone marrow suppression  -Rheumatoid, infectious/septic, or other acute inflammatory arthritis.  -Severe renal function impairment.  -Active or recurrent infection.  -Any surgical or underlying hepatic, haematological, pulmonary, infectious, or gastrointestinal condition.  -Significant medical problems (e.g. uncontrolled hypertension, congestive heart disease, uncontrolled diabetes type 1 or type 2, thyroid disease; history of malignancy of any organ system within the preceding 5 years).  -History of organ transplantation.  -Pregnant or nursing (lactating) women.  -Women who are physiologically capable of becoming pregnant unless using an acceptable method of contraception.  -History of alcohol or drug abuse within 12 months of randomisation. | -Safety assessments were performed at scheduled visits (2, 4, 8, 12, 16, 20 and 24 weeks) and included collection of blood samples for assessment of inflammation, haematology and immunogenicity.  -Adverse events were reported by patients throughout the study. |
| Schnebel 1988 [[40](#_ENREF_40)] | -History of low back pain with or without leg pain, of less than 3 months duration | -Not specified | -Assessment method of AEs not specified |
| Seideman 1987 [[30](#_ENREF_30)] | -Aged 20 to 65 years.  -Confirmed psoriatic skin lesions with diagnosis established clinically and by skin biopsy. | -Confirmed hepatic or renal symptoms.  -Previous gastrointestinal intolerance to drugs. | -A questionnaire used to register adverse events during study visits at one-month intervals. |
| Tardif 2019 [[37](#_ENREF_37)] | -Adult patients.  -Had a myocardial infarction within 30 days before enrolment.  -Had completed any planned percutaneous revascularization procedures.  -Were treated according to national guidelines that included the intensive use of statins. | -Severe heart failure.  -A left ventricular ejection fraction of less than 35%, stroke within the previous 3 months.  -A type 2 index myocardial infarction.  -Coronary-bypass surgery either within the previous 3 years or planned.  -A history of non-cutaneous cancer within the previous 3 years.  -Inflammatory bowel disease or chronic diarrhea.  -Neuromuscular disease or a non-transient creatine kinase level that was greater than three times the upper limit of the normal range (unless due to infarction).  -Clinically significant non-transient hematologic abnormalities.  -Severe renal disease with a serum creatinine level that was greater than two times the upper limit of the normal range.  -Severe hepatic disease.  -Drug or alcohol abuse.  -Current or planned long-term systemic glucocorticoid therapy.  -A history of clinically significant sensitivity to colchicine. | -All adverse events reported were judged by the investigator to be related to the study drug.  -Laboratory tests were undertaken to determine hematological abnormalities. |
| Terkeltaub 2010 [[22](#_ENREF_22)] | -Male and postmenopausal female patients.  -Aged 18 years or older  -Diagnosis of gout according got ACR criteria.  -Had 2 gout flares within the prior 12 months. | -Not specified | -The patient was specifically asked about the presence of nausea, vomiting, diarrhea, and abdominal pain every time the patient rated pain, along with an open-ended question about other AEs. -The patient was also supplied with a standardized diary to track pain, symptoms, AEs, and rescue medication use. |
| Wang 2014 [[23](#_ENREF_23)] | -Aged 18 years or older.  -Newly diagnosed gout according to the ACR criteria.  -Onset of the disease duration of less than 48 hours. | -Rheumatoid arthritis, septic arthritis, traumatic arthritis, etc  -Had gout in the intermittent period or with tophi  -Taking the following drugs: diuretics, pyrazinamide, aspirin, etc.  -Pregnant or breast-feeding women  -Suffering from cardiovascular and cerebral vascular disease, and severe trauma or had undergone surgery.  -Had severe infections.  -Were suffering from hepatobiliary disease or whose aspartate aminotransferase and alanine aminotransferase were 2 times higher than upper limit of normal.  -Had serum creatinine levels greater than the upper limit of normal.  -Had severe chronic gastrointestinal disease.  -Had hematological diseases or endocrine system diseases.  -Were undergoing cancer treatment.  -Were receiving steroid therapy.  -Were allergic to those known ingredients in the Chuanhu anti-gout mixture. | -Adverse events were recorded in the patient diaries. |
| Yurdakul 2001 [[29](#_ENREF_29)] | -Aged 18–35 years of age  -Have active disease  -Have a disease duration of >2 years.  -Live at a reasonable traveling distance from study centre. | -Received immunosuppressive agents, steroids, or colchicine within the preceding 6 months.  -Organ involvement requiring immunosuppression.  -Eye disease, especially with retinal involvement. | -Adverse effects were recorded by questioning patients regarding loss of appetite, nausea, abdominal pain, and diarrhea or any other symptom volunteered by the patient at each visit. |

| **Supplementary Table 2.** Frequency of any adverse event reported in colchicine and comparator groups | | | | | | | | | | | | | | | | | | | | |
| --- | --- | --- | --- | --- | --- | --- | --- | --- | --- | --- | --- | --- | --- | --- | --- | --- | --- | --- | --- | --- |
|  |  | | **Number of participants with adverse events** | | | | | | | | | | | | | | | | | |
|  | **Number of participants randomised** | | **Diarrhoea** | | **Any GI event** | | **Any liver event** | | **Any hematologic event** | | **Any muscle-related event** | | **Any sensory event** | | **Any infectious event** | | **Death** | | **Any adverse event** | |
|  | **Colchicine** | **Comparator** | **Colchicine** | **Comparator** | **Colchicine** | **Comparator** | **Colchicine** | **Comparator** | **Colchicine** | **Comparator** | **Colchicine** | **Comparator** | **Colchicine** | **Comparator** | **Colchicine** | **Comparator** | **Colchicine** | **Comparator** | **Colchicine** | **Comparator** |
| Aran 2011 [[24](#_ENREF_24)] | 31 | 30 | 1 | 0 | 1 | 0 | NR | NR | NR | NR | NR | NR | NR | NR | NR | NR | NR | NR | 1 | 0 |
| Batezzati 2001 [[6](#_ENREF_6)] | 22 | 22 | 1 | 0 | 1 | 0 | NR | NR | NR | NR | NR | NR | NR | NR | NR | NR | NR | NR | 1 | 0 |
| Bessissow 2018 [[36](#_ENREF_36)] | 49 | 51 | 5 | 1 | NR | NR | NR | NR | NR | NR | NR | NR | NR | NR | 6 | 8 | 0 | 0 | NR | NR |
| Borstad 2004 [[20](#_ENREF_20)] | 21 | 22 | 8 | 1 | 8 | 1 | NR | NR | NR | NR | NR | NR | NR | NR | NR | NR | NR | NR | 9 | 8 |
| Cohen 1991 [[33](#_ENREF_33)] | 8 | 8 | NR | NR | NR | NR | NR | NR | 0 | 0 | NR | NR | NR | NR | NR | NR | NR | NR | NR | NR |
| Cortez-Pinto 2002 [[7](#_ENREF_7)] | 29 | 26 | 7 | 0 | 7 | 0 | NR | NR | NR | NR | NR | NR | NR | NR | NR | NR | NR | NR | 7 | 0 |
| Das 2002 [[25](#_ENREF_25)] | 19 | 20 | NR | NR | NR | NR | NR | NR | NR | NR | NR | NR | NR | NR | NR | NR | 0 | 0 | NR | NR |
| Davatchi 2009 [[27](#_ENREF_27)] | 136 | 146 | 4 | 3 | 4 | 3 | 2 | 0 | 0 | 1 | NR | NR | 0 | 1 | 1 | 1 | NR | NR | 9 | 9 |
| Deftereos 2013 [[34](#_ENREF_34)] | 112 | 110 | NR | NR | 16 | 7 | 0 | 0 | 0 | 0 | 15 | 10 | NR | NR | NR | NR | NR | NR | NR | NR |
| Demidowich 2019 [[35](#_ENREF_35)] | 21 | 19 | NR | NR | 13 | 14 | 7 | 4 | 2 | 1 | 4 | 6 | NR | NR | 6 | 11 | NR | NR | NR | NR |
| Dinarello 1974 [[12](#_ENREF_12)] | 11 | 11 | NR | NR | NR | NR | 0 | 0 | 0 | 0 | NR | NR | NR | NR | NR | NR | NR | NR | NR | NR |
| Fish 1997 [[38](#_ENREF_38)] | 35 | 36 | NR | NR | 9 | 6 | NR | NR | NR | NR | NR | NR | NR | NR | NR | NR | NR | NR | 9 | 6 |
| Imazio 2010 [[31](#_ENREF_31)] | 180 | 180 | NR | NR | 16 | 8 | 0 | 0 | 0 | 0 | 0 | 1 | NR | NR | NR | NR | NR | NR | 16 | 9 |
| Imazio 2011[[16](#_ENREF_16)] | 60 | 60 | NR | NR | 4 | 3 | 0 | 1 | NR | NR | 0 | 0 | NR | NR | NR | NR | NR | NR | 4 | 4 |
| Imazio 2013 [[17](#_ENREF_17)] | 120 | 120 | NR | NR | 11 | 10 | 2 | 1 | NR | NR | 0 | 0 | NR | NR | NR | NR | NR | NR | 14 | 12 |
| Imazio 2014a [[32](#_ENREF_32)] | 180 | 180 | NR | NR | 26 | 12 | 1 | 2 | NR | NR | 0 | 0 | NR | NR | NR | NR | NR | NR | 36 | 21 |
| Imazio 2014b [[18](#_ENREF_18)] | 120 | 120 | NR | NR | 9 | 9 | 3 | 1 | NR | NR | 1 | 0 | NR | NR | NR | NR | NR | NR | 14 | 10 |
| Kaplan 1986 [[8](#_ENREF_8)] | 30 | 30 | 4 | 0 | 4 | 0 | NR | NR | NR | NR | NR | NR | NR | NR | NR | NR | NR | NR | 5 | 1 |
| Kershenobich 1979 [[9](#_ENREF_9)] | 23 | 20 | 2 | 0 | 2 | 0 | NR | NR | NR | NR | NR | NR | NR | NR | NR | NR | NR | NR | 2 | 0 |
| Kershenobich 1988 [[10](#_ENREF_10)] | 54 | 46 | 9 | 0 | 9 | 0 | NR | NR | NR | NR | NR | NR | NR | NR | NR | NR | NR | NR | 11 | 1 |
| Leung 2018 [[26](#_ENREF_26)] | 54 | 55 | 19 | 12 | 19 | 12 | 4 | 1 | NR | NR | 12 | 8 | 2 | 2 | 17 | 10 | 0 | 0 | 42 | 35 |
| Liebenburg 2016[[19](#_ENREF_19)] | 19 | 14 | 11 | 0 | 11 | 0 | 1 | 0 | NR | NR | NR | NR | NR | NR | NR | NR | NR | NR | 12 | 0 |
| Masuda 1989 [[28](#_ENREF_28)] | 49 | 47 | NR | NR | NR | NR | NR | NR | NR | NR | NR | NR | NR | NR | NR | NR | NR | NR | NR | NR |
| McKendry 1993 [[13](#_ENREF_13)] | 25 | 25 | NR | NR | NR | NR | NR | NR | NR | NR | 5 | 4 | NR | NR | NR | NR | NR | NR | 14 | 4 |
| Olsson 1995 [[11](#_ENREF_11)] | 44 | 40 | 1 | 0 | 1 | 0 | NR | NR | 0 | 0 | NR | NR | NR | NR | NR | NR | NR | NR | 1 | 0 |
| Pakfetrat 2010 [[39](#_ENREF_39)] | 17 | 17 | NR | NR | 8 | 0 | NR | NR | NR | NR | NR | NR | NR | NR | NR | NR | NR | NR | 9 | 2 |
| Paulus 1974 [[21](#_ENREF_21)] | 29 | 23 | 12 | 6 | 12 | 6 | 1 | 0 | NR | NR | NR | NR | NR | NR | 11 | 5 | NR | NR | 15 | 8 |
| Roche 1995 [[14](#_ENREF_14)] | 16 | 16 | 7 | 2 | 7 | 2 | NR | NR | NR | NR | NR | NR | NR | NR | NR | NR | NR | NR | NR | NR |
| Schlesinger 2011[[15](#_ENREF_15)] | 108 | 324 | NR | NR | 2 | 7 | 1 | 5 | NR | NR | NR | NR | NR | NR | 13 | 58 | NR | NR | 58 | 175 |
| Schnebel 1988 [[40](#_ENREF_40)] | 17 | 17 | 9 | 4 | 9 | 4 | NR | NR | NR | NR | NR | NR | NR | NR | NR | NR | NR | NR | 10 | 3 |
| Seideman 1987 [[30](#_ENREF_30)] | 15 | 15 | NR | NR | 7 | 0 | NR | NR | NR | NR | NR | NR | NR | NR | NR | NR | NR | NR | 7 | 0 |
| Tardif 2019 [[37](#_ENREF_37)] | 2366 | 2379 | 225 | 208 | 408 | 414 | NR | NR | 14 | 10 | NR | NR | NR | NR | 51 | 38 | NR | NR | 408 | 414 |
| Terkeltaub 2010 [[22](#_ENREF_22)] (low dose) | 74 | 59 | 17 | 8 | 19 | 12 | NR | NR | NR | NR | NR | NR | NR | NR | NR | NR | 0 | 0 | 27 | 16 |
| Terkeltaub 2010 [[22](#_ENREF_22)] (high dose) | 52 | 59 | 40 | 8 | 40 | 12 | NR | NR | NR | NR | NR | NR | NR | NR | NR | NR | 0 | 0 | 40 | 16 |
| Terkeltaub 2010 [[22](#_ENREF_22)] (total) | 126 | 59 | 57 | 8 | 59 | 12 | NR | NR | NR | NR | NR | NR | NR | NR | NR | NR | 0 | 0 | 67 | 16 |
| Wang 2014 [[23](#_ENREF_23)] | 88 | 88 | 21 | 1 | 21 | 1 | NR | NR | NR | NR | NR | NR | NR | NR | NR | NR | NR | NR | 25 | 2 |
| Yurdakul 2001 [[29](#_ENREF_29)] | 58 | 58 | 17 | 16 | 17 | 17 | NR | NR | NR | NR | NR | NR | NR | NR | NR | NR | NR | NR | 39 | 44 |
| * = active-comparator studies; NR = not-reported; GI = gastrointestinal | | | | | | | | | | | | | | | | | | | | |

| **Supplementary Table 3.** Meta-analysis results showing pooled risk ratio of adverse events between colchicine and pooled comparator groups for studies not involving participants with liver diseases | | | | | | |
| --- | --- | --- | --- | --- | --- | --- |
|  | **N. studies** | **n/N, % (95% CI) participants** | | **Pooled risk ratio (95% CI)** | **I^2^ (p-value)** | **Overall effect, Z (p-value)** |
|  |  | **Colchicine** | **Comparator** |  |  |  |
| Any event | 21 | 818/3805, 21.5% (20.2, 22.8) | 782/3954, 19.8% (18.6, 21.0) | 1.37 (1.14, 1.65) | 60% (<0.001) | 3.31 (<0.001) |
| Diarrhoea | 13 | 396/3010, 13.2% (12.0, 14.4) | 262/2958, 8.9% (7.9, 9.9) | 2.14 (1.40, 3.26) | 64% (0.001) | 3.53 (<0.001) |
| Gastrointestinal | 23 | 705/3929, 17.9% (16.8, 19.2) | 552/4029, 13.7% (12.7, 14.8) | 1.60 (1.22, 2.10) | 55% (0.001) | 3.39 (<0.001) |
|  | | | | | | |

| **Supplementary Table 4**. Number of participants in colchicine and comparator groups with adverse events related to gastrointestinal, liver and hematologic events | | | | | | | | | | | | | | | | | | | | | | | | | | | | | | | | | | | | | | | | | | | | | | | | | | | | | | | | | | | | | | | | | | | | | | | | | | | | | | | | | | | | | | | | | | | | | | |
| --- | --- | --- | --- | --- | --- | --- | --- | --- | --- | --- | --- | --- | --- | --- | --- | --- | --- | --- | --- | --- | --- | --- | --- | --- | --- | --- | --- | --- | --- | --- | --- | --- | --- | --- | --- | --- | --- | --- | --- | --- | --- | --- | --- | --- | --- | --- | --- | --- | --- | --- | --- | --- | --- | --- | --- | --- | --- | --- | --- | --- | --- | --- | --- | --- | --- | --- | --- | --- | --- | --- | --- | --- | --- | --- | --- | --- | --- | --- | --- | --- | --- | --- | --- | --- | --- | --- | --- | --- | --- | --- | --- | --- | --- | --- |
|  | | | GI events | | | | | | | | | | | | | | | | | | | | | | | | | | | | | | | | | | | | | | | | | | | | | | | | Liver events | | | | | | | | | | | | | | | | | | Hematologic events | | | | | | | | | | | | | | | | | | | | | | | | |  |
|  | | | Diarrhoea | | | Nausea | | Vomiting | | | | Diarrhoea or nausea | | | GI distress or diarrhoea | | | | Diarrhoea, nausea &/or vomiting | | | Abdominal pain | | | | GI symptoms/effects | | | | Loss of appetite | | | Bloating | | | | Constipation | | | Melena/ hemorrhage | | | | Peptic ulcer | | | | **Any GI event** | | | Hepatotoxicity | | | | Hepatic abnormalities | | | Hepatitis | | | | Increased liver enzymes | | | | **Any liver event** | | | Anaemia | | | | Hematotoxicity | | | Hematologic abnormalities | | | | Bone marrow toxicity | | | | Leukopenia | | | Purpura | | | | **Any hematologic event** | | |  |
|  | |  | Colchicine | Comparator | | Colchicine | Comparator | Colchicine | | Comparator | | Colchicine | Comparator | | Colchicine | | Comparator | | Colchicine | | Comparator | Colchicine | | Comparator | | Colchicine | | Comparator | | Colchicine | Comparator | | Colchicine | | Comparator | | Colchicine | | Comparator | Colchicine | | Comparator | | Colchicine | | Comparator | | **Colchicine** | **Comparator** | | Colchicine | | Comparator | | Colchicine | | Comparator | Colchicine | | Comparator | | Colchicine | | Comparator | | **Colchicine** | **Comparator** | | Colchicine | | Comparator | | Colchicine | | Comparator | Colchicine | | Comparator | | Colchicine | | Comparator | | Colchicine | Comparator | | Colchicine | | Comparator | | **Colchicine** | | **Comparator** |  |
| Aran 2011 [[24](#_ENREF_24)] | | | 1 | 0 | | 1 | 0 | 1 | | 0 | | NR | NR | | NR | | NR | | NR | | NR | NR | | NR | | NR | | NR | | NR | NR | | NR | | NR | | NR | | NR | NR | | NR | | NR | | NR | | **1** | **0** | | NR | | NR | | NR | | NR | NR | | NR | | NR | | NR | | **NR** | **NR** | | NR | | NR | | NR | | NR | NR | | NR | | NR | | NR | | NR | NR | | NR | | NR | | **NR** | | **NR** |  |
| Batezzati 2001 [[6](#_ENREF_6)] | | | 0 | 1 | | NR | NR | NR | | NR | | NR | NR | | NR | | NR | | NR | | NR | NR | | NR | | NR | | NR | | NR | NR | | NR | | NR | | NR | | NR | NR | | NR | | NR | | NR | | **0** | **1** | | NR | | NR | | NR | | NR | NR | | NR | | NR | | NR | | **NR** | **NR** | | NR | | NR | | NR | | NR | NR | | NR | | NR | | NR | | NR | NR | | NR | | NR | | **NR** | | **NR** |  |
| Bessissow 2018 [[36](#_ENREF_36)] | | | 5 | 1 | | NR | NR | NR | | NR | | NR | NR | | NR | | NR | | NR | | NR | NR | | NR | | NR | | NR | | NR | NR | | NR | | NR | | NR | | NR | NR | | NR | | NR | | NR | | **5** | **1** | | NR | | NR | | NR | | NR | NR | | NR | | NR | | NR | | NR | NR | | NR | | NR | | NR | | NR | NR | | NR | | NR | | NR | | NR | NR | | NR | | NR | | NR | | NR |  |
| Borstad 2004 [[20](#_ENREF_20)] | | | 8 | 1 | | NR | NR | NR | | NR | | NR | NR | | NR | | NR | | NR | | NR | NR | | NR | | NR | | NR | | NR | NR | | NR | | NR | | NR | | NR | NR | | NR | | NR | | NR | | **8** | **1** | | NR | | NR | | NR | | NR | NR | | NR | | NR | | NR | | **NR** | **NR** | | NR | | NR | | NR | | NR | NR | | NR | | NR | | NR | | NR | NR | | NR | | NR | | **NR** | | **NR** |  |
| Cohen 1991 [[33](#_ENREF_33)] | | | NR | NR | | NR | NR | NR | | NR | | NR | NR | | NR | | NR | | NR | | NR | NR | | NR | | NR | | NR | | NR | NR | | NR | | NR | | NR | | NR | NR | | NR | | NR | | NR | | **NR** | **NR** | | NR | | NR | | NR | | NR | NR | | NR | | NR | | NR | | **NR** | **NR** | | 0 | | 0 | | NR | | NR | NR | | NR | | NR | | NR | | 0 | 0 | | NR | | NR | | **0** | | **0** |  |
| Cortez-Pinto 2002 [[7](#_ENREF_7)] | | | 7 | 0 | | NR | NR | NR | | NR | | NR | NR | | NR | | NR | | NR | | NR | NR | | NR | | NR | | NR | | NR | NR | | NR | | NR | | NR | | NR | NR | | NR | | NR | | NR | | **7** | **0** | | NR | | NR | | NR | | NR | NR | | NR | | NR | | NR | | **NR** | **NR** | | NR | | NR | | NR | | NR | NR | | NR | | NR | | NR | | NR | NR | | NR | | NR | | **NR** | | **NR** |  |
| Das 2002 [[25](#_ENREF_25)] | | | NR | NR | | NR | NR | NR | | NR | | NR | NR | | NR | | NR | | NR | | NR | NR | | NR | | NR | | NR | | NR | NR | | NR | | NR | | NR | | NR | NR | | NR | | NR | | NR | | **NR** | **NR** | | NR | | NR | | NR | | NR | NR | | NR | | NR | | NR | | **NR** | **NR** | | NR | | NR | | NR | | NR | NR | | NR | | NR | | NR | | NR | NR | | NR | | NR | | **NR** | | **NR** |  |
| Davatchi 2009 [[27](#_ENREF_27)] | | | 4 | 3 | | NR | NR | NR | | NR | | NR | NR | | NR | | NR | | NR | | NR | 0 | | 1 | | NR | | NR | | NR | NR | | NR | | NR | | NR | | NR | NR | | NR | | NR | | NR | | **4** | **3** | | NR | | NR | | NR | | NR | NR | | NR | | 2 | | 0 | | **2** | **0** | | 0 | | 1 | | NR | | NR | NR | | NR | | NR | | NR | | NR | NR | | 0 | | 1 | | **0** | | **1** |  |
| Deftereos 2013 [[34](#_ENREF_34)] | | | NR | NR | | NR | NR | NR | | NR | | 16 | 7 | | NR | | NR | | NR | | NR | NR | | NR | | NR | | NR | | NR | NR | | NR | | NR | | NR | | NR | NR | | NR | | NR | | NR | | **16** | **7** | | 0 | | 0 | | NR | | NR | NR | | NR | | NR | | NR | | **0** | **0** | | NR | | NR | | 0 | | 0 | NR | | NR | | NR | | NR | | NR | NR | | NR | | NR | | **0** | | **0** |  |
| Demidowich 2019 [[35](#_ENREF_35)] | | | NR | NR | | NR | NR | NR | | NR | | NR | NR | | NR | | NR | | NR | | NR | NR | | NR | | 13 | | 14 | | NR | NR | | NR | | NR | | NR | | NR | NR | | NR | | NR | | NR | | **13** | **14** | | NR | | NR | | NR | | NR | NR | | NR | | 7 | | 4 | | **7** | **4** | | 0 | | 1 | | NR | | NR | 2 | | 0 | | NR | | NR | | 2 | 0 | | NR | | NR | | **2** | | **1** |  |
| Dinarello 1974 [[12](#_ENREF_12)] | | | NR | NR | | NR | NR | NR | | NR | | NR | NR | | NR | | NR | | NR | | NR | NR | | NR | | NR | | NR | | NR | NR | | NR | | NR | | NR | | NR | NR | | NR | | NR | | NR | | **NR** | **NR** | | NR | | NR | | 0 | | 0 | NR | | NR | | NR | | NR | | **0** | **0** | | NR | | NR | | NR | | NR | 0 | | 0 | | NR | | NR | | NR | NR | | NR | | NR | | **0** | | **0** |  |
| Fish 1997 [[38](#_ENREF_38)] | | | NR | NR | | NR | NR | NR | | NR | | NR | NR | | NR | | NR | | NR | | NR | NR | | NR | | 9 | | 6 | | NR | NR | | NR | | NR | | NR | | NR | NR | | NR | | NR | | NR | | **9** | **6** | | NR | | NR | | NR | | NR | NR | | NR | | NR | | NR | | **NR** | **NR** | | NR | | NR | | NR | | NR | NR | | NR | | NR | | NR | | NR | NR | | NR | | NR | | **NR** | | **NR** |  |
| Imazio 2010 [[31](#_ENREF_31)] | | | NR | NR | | NR | NR | NR | | NR | | NR | NR | | NR | | NR | | NR | | NR | NR | | NR | | 16 | | 8 | | 0 | 0 | | NR | | NR | | NR | | NR | NR | | NR | | NR | | NR | | **16** | **8** | | 0 | | 0 | | NR | | NR | NR | | NR | | NR | | NR | | **0** | **0** | | NR | | NR | | NR | | NR | NR | | NR | | 0 | | 0 | | NR | NR | | NR | | NR | | **0** | | **0** |  |
| Imazio 2011[[16](#_ENREF_16)] | | | NR | NR | | NR | NR | NR | | NR | | NR | NR | | NR | | NR | | NR | | NR | NR | | NR | | 4 | | 3 | | NR | NR | | NR | | NR | | NR | | NR | NR | | NR | | NR | | NR | | **4** | **3** | | 0 | | 1 | | NR | | NR | NR | | NR | | NR | | NR | | **0** | **1** | | NR | | NR | | NR | | NR | NR | | NR | | NR | | NR | | NR | NR | | NR | | NR | | **NR** | | **NR** |  |
| Imazio 2013 [[17](#_ENREF_17)] | | | NR | NR | | NR | NR | NR | | NR | | NR | NR | | NR | | NR | | NR | | NR | NR | | NR | | 11 | | 10 | | NR | NR | | NR | | NR | | NR | | NR | NR | | NR | | NR | | NR | | **11** | **10** | | 2 | | 1 | | NR | | NR | NR | | NR | | NR | | NR | | **2** | **1** | | NR | | NR | | NR | | NR | NR | | NR | | NR | | NR | | NR | NR | | NR | | NR | | **NR** | | **NR** |  |
| Imazio 2014a [[32](#_ENREF_32)] | | | NR | NR | | NR | NR | NR | | NR | | NR | NR | | NR | | NR | | NR | | NR | NR | | NR | | 26 | | 12 | | NR | NR | | NR | | NR | | NR | | NR | NR | | NR | | NR | | NR | | **26** | **12** | | 1 | | 2 | | NR | | NR | NR | | NR | | NR | | NR | | **1** | **2** | | NR | | NR | | NR | | NR | NR | | NR | | NR | | NR | | NR | NR | | NR | | NR | | **NR** | | **NR** |  |
| Imazio 2014b [[18](#_ENREF_18)] | | | NR | NR | | NR | NR | NR | | NR | | NR | NR | | NR | | NR | | NR | | NR | NR | | NR | | 9 | | 9 | | NR | NR | | NR | | NR | | NR | | NR | NR | | NR | | NR | | NR | | **9** | **9** | | 3 | | 1 | | NR | | NR | NR | | NR | | NR | | NR | | **3** | **1** | | NR | | NR | | NR | | NR | NR | | NR | | NR | | NR | | NR | NR | | NR | | NR | | **NR** | | **NR** |  |
| Kaplan 1986 [[8](#_ENREF_8)] | | | 4 | 0 | | NR | NR | NR | | NR | | NR | NR | | NR | | NR | | NR | | NR | NR | | NR | | NR | | NR | | NR | NR | | NR | | NR | | NR | | NR | NR | | NR | | NR | | NR | | **4** | **0** | | NR | | NR | | NR | | NR | NR | | NR | | NR | | NR | | **NR** | **NR** | | NR | | NR | | NR | | NR | NR | | NR | | NR | | NR | | NR | NR | | NR | | NR | | **NR** | | **NR** |  |
| Kershenobich 1979 [[9](#_ENREF_9)] | | | 2 | 0 | | NR | NR | NR | | NR | | NR | NR | | NR | | NR | | NR | | NR | NR | | NR | | NR | | NR | | NR | NR | | NR | | NR | | NR | | NR | NR | | NR | | NR | | NR | | **2** | **0** | | NR | | NR | | NR | | NR | NR | | NR | | NR | | NR | | **NR** | **NR** | | NR | | NR | | NR | | NR | NR | | NR | | NR | | NR | | NR | NR | | NR | | NR | | **NR** | | **NR** |  |
| Kershenobich 1988 [[10](#_ENREF_10)] | | | 9 | 0 | | NR | NR | NR | | NR | | NR | NR | | NR | | NR | | NR | | NR | NR | | NR | | NR | | NR | | NR | NR | | NR | | NR | | NR | | NR | NR | | NR | | 2 | | 1 | | **9** | **0** | | NR | | NR | | NR | | NR | NR | | NR | | NR | | NR | | **NR** | **NR** | | NR | | NR | | NR | | NR | NR | | NR | | NR | | NR | | NR | NR | | NR | | NR | | **NR** | | **NR** |  |
| Leung 2018 [[26](#_ENREF_26)] | | | 19 | 12 | | NR | NR | NR | | NR | | NR | NR | | NR | | NR | | NR | | NR | NR | | NR | | NR | | NR | | NR | NR | | 3 | | 3 | | NR | | NR | NR | | NR | | NR | | NR | | **19** | **12** | | NR | | NR | | NR | | NR | NR | | NR | | 4 | | 1 | | **4** | **1** | | NR | | NR | | NR | | NR | NR | | NR | | NR | | NR | | NR | NR | | NR | | NR | | **NR** | | **NR** |  |
| Liebenburg 2016[[19](#_ENREF_19)] | | | 11 | 0 | | NR | NR | NR | | NR | | NR | NR | | NR | | NR | | NR | | NR | NR | | NR | | NR | | NR | | NR | NR | | NR | | NR | | NR | | NR | NR | | NR | | NR | | NR | | **11** | **0** | | NR | | NR | | NR | | NR | 1 | | 0 | | NR | | NR | | **1** | **0** | | NR | | NR | | NR | | NR | NR | | NR | | NR | | NR | | NR | NR | | NR | | NR | | **NR** | | **NR** |  |
| Masuda 1989 [[28](#_ENREF_28)] | | | NR | NR | | NR | NR | NR | | NR | | NR | NR | | NR | | NR | | NR | | NR | NR | | NR | | NR | | NR | | NR | NR | | NR | | NR | | NR | | NR | NR | | NR | | NR | | NR | | **NR** | **NR** | | NR | | NR | | NR | | NR | NR | | NR | | NR | | NR | | **NR** | **NR** | | NR | | NR | | NR | | NR | NR | | NR | | NR | | NR | | NR | NR | | NR | | NR | | **NR** | | **NR** |  |
| McKendry 1993 [[13](#_ENREF_13)] | | | NR | NR | | NR | NR | NR | | NR | | NR | NR | | NR | | NR | | NR | | NR | NR | | NR | | NR | | NR | | NR | NR | | NR | | NR | | NR | | NR | NR | | NR | | NR | | NR | | **NR** | **NR** | | NR | | NR | | NR | | NR | NR | | NR | | NR | | NR | | **NR** | **NR** | | NR | | NR | | NR | | NR | NR | | NR | | NR | | NR | | NR | NR | | NR | | NR | | **NR** | | **NR** |  |
| Olsson 1995 [[11](#_ENREF_11)] | | | 1 | 0 | | NR | NR | NR | | NR | | NR | NR | | NR | | NR | | NR | | NR | NR | | NR | | NR | | NR | | NR | NR | | NR | | NR | | NR | | NR | NR | | NR | | NR | | NR | | **1** | **0** | | NR | | NR | | NR | | NR | NR | | NR | | NR | | NR | | **NR** | **NR** | | NR | | NR | | NR | | NR | 0 | | 0 | | NR | | NR | | NR | NR | | NR | | NR | | **0** | | **0** |  |
| Pakfetrat 2010 [[39](#_ENREF_39)] | | | NR | NR | | NR | NR | NR | | NR | | NR | NR | | NR | | NR | | NR | | NR | NR | | NR | | 8 | | 0 | | NR | NR | | NR | | NR | | NR | | NR | NR | | NR | | NR | | NR | | **8** | **0** | | NR | | NR | | NR | | NR | NR | | NR | | NR | | NR | | **NR** | **NR** | | NR | | NR | | NR | | NR | NR | | NR | | NR | | NR | | NR | NR | | NR | | NR | | **NR** | | **NR** |  |
| Paulus 1974 [[21](#_ENREF_21)] | | | 12 | 6 | | NR | NR | NR | | NR | | NR | NR | | NR | | NR | | NR | | NR | NR | | NR | | NR | | NR | | NR | NR | | NR | | NR | | NR | | NR | NR | | NR | | NR | | NR | | **12** | **6** | | NR | | NR | | NR | | NR | NR | | NR | | 1 | | 0 | | **1** | **0** | | NR | | NR | | NR | | NR | NR | | NR | | NR | | NR | | NR | NR | | NR | | NR | | **NR** | | **NR** |  |
| Roche 1995 [[14](#_ENREF_14)] | | | 7 | 2 | | NR | NR | NR | | NR | | NR | NR | | NR | | NR | | NR | | NR | 5 | | 2 | | NR | | NR | | NR | NR | | NR | | NR | | NR | | NR | NR | | NR | | NR | | NR | | **7** | **2** | | NR | | NR | | NR | | NR | NR | | NR | | NR | | NR | | **NR** | **NR** | | NR | | NR | | NR | | NR | NR | | NR | | NR | | NR | | NR | NR | | NR | | NR | | **NR** | | **NR** |  |
| Schlesinger 2011[[15](#_ENREF_15)] | | | NR | NR | | 1 | 7 | NR | | NR | | NR | NR | | NR | | NR | | NR | | NR | NR | | NR | | NR | | NR | | NR | NR | | NR | | NR | | 2 | | 0 | NR | | NR | | NR | | NR | | **2** | **7** | | NR | | NR | | NR | | NR | NR | | NR | | 1 | | 5 | | **1** | **5** | | NR | | NR | | NR | | NR | NR | | NR | | NR | | NR | | NR | NR | | NR | | NR | | **NR** | | **NR** |  |
| Schnebel 1988 [[40](#_ENREF_40)] | | | 9 | 4 | | 0 | 1 | 1 | | 0 | | NR | NR | | NR | | NR | | NR | | NR | NR | | NR | | NR | | NR | | NR | NR | | NR | | NR | | NR | | NR | NR | | NR | | NR | | NR | | **9** | **4** | | NR | | NR | | NR | | NR | NR | | NR | | NR | | NR | | **NR** | **NR** | | NR | | NR | | NR | | NR | NR | | NR | | NR | | NR | | NR | NR | | NR | | NR | | **NR** | | **NR** |  |
| Seideman 1987 [[30](#_ENREF_30)] | | | NR | NR | | NR | NR | NR | | NR | | NR | NR | | 7 | | 0 | | NR | | NR | NR | | NR | | NR | | NR | | NR | NR | | NR | | NR | | NR | | NR | NR | | NR | | NR | | NR | | **7** | **0** | | NR | | NR | | NR | | NR | NR | | NR | | NR | | NR | | **NR** | **NR** | | NR | | NR | | NR | | NR | NR | | NR | | NR | | NR | | NR | NR | | NR | | NR | | **NR** | | **NR** |  |
| Tardif 2019 [[37](#_ENREF_37)] | | | 225 | 414 | | 43 | 24 | NR | | NR | | NR | NR | | NR | | NR | | NR | | NR | NR | | NR | | 408 | | 414 | | NR | NR | | NR | | NR | | NR | | NR | 7 | | 5 | | NR | | NR | | **408** | **414** | | NR | | NR | | NR | | NR | NR | | NR | | NR | | NR | | NR | NR | | 14 | | 10 | | NR | | NR | 3 | | 7 | | NR | | NR | | 2 | 3 | | NR | | NR | | **14** | | **10** |  |
| Terkeltaub 2010 [[22](#_ENREF_22)] | | Low dose | 17 | 8 | | 3 | 3 | 0 | | 0 | | NR | NR | | NR | | NR | | 19 | | 12 | NR | | NR | | NR | | NR | | NR | NR | | NR | | NR | | NR | | NR | 0 | | 0 | | NR | | NR | | **19** | **12** | | NR | | NR | | NR | | NR | NR | | NR | | NR | | NR | | **NR** | **NR** | | NR | | NR | | NR | | NR | NR | | NR | | NR | | NR | | NR | NR | | NR | | NR | | **NR** | | **NR** |  |
|  |  | High dose | 40 | 8 | | 9 | 3 | 9 | | 0 | | NR | NR | | NR | | NR | | 14 | | 12 | NR | | NR | | NR | | NR | | NR | NR | | NR | | NR | | NR | | NR | 1 | | 0 | | NR | | NR | | **40** | **12** | | NR | | NR | | NR | | NR | NR | | NR | | NR | | NR | | **NR** | **NR** | | NR | | NR | | NR | | NR | NR | | NR | | NR | | NR | | NR | NR | | NR | | NR | | **NR** | | **NR** |  |
|  |  | Total | 57 | 8 | | 12 | 3 | 9 | | 0 | | NR | NR | | NR | | NR | | 33 | | 12 | NR | | NR | | NR | | NR | | NR | NR | | NR | | NR | | NR | | NR | 1 | | 0 | | NR | | NR | | **59** | **12** | | NR | | NR | | NR | | NR | NR | | NR | | NR | | NR | | **NR** | **NR** | | NR | | NR | | NR | | NR | NR | | NR | | NR | | NR | | NR | NR | | NR | | NR | | **NR** | | **NR** |  |
| Wang 2014 [[23](#_ENREF_23)] | | | 21 | 1 | | 3 | 1 | 1 | | 0 | | NR | NR | | NR | | NR | | NR | | NR | NR | | NR | | NR | | NR | | NR | NR | | NR | | NR | | NR | | NR | NR | | NR | | NR | | NR | | **21** | **1** | | NR | | NR | | NR | | NR | NR | | NR | | NR | | NR | | **NR** | **NR** | | NR | | NR | | NR | | NR | NR | | NR | | NR | | NR | | NR | NR | | NR | | NR | | **NR** | | **NR** |  |
| Yurdakul 2001 [[29](#_ENREF_29)] | | | 17 | 16 | | 10 | 17 | NR | | NR | | NR | NR | | NR | | NR | | NR | | NR | 16 | | 12 | | NR | | NR | | 15 | 15 | | NR | | NR | | NR | | NR | NR | | NR | | NR | | NR | | **17** | **17** | | NR | | NR | | NR | | NR | NR | | NR | | NR | | NR | | **NR** | **NR** | | NR | | NR | | NR | | NR | NR | | NR | | NR | | NR | | NR | NR | | NR | | NR | | **NR** | | **NR** |  |
| **Supplementary Table 5**. Number of participants in colchicine and comparator groups with adverse events related to muscle, sensory, and infectious events | | | | | | | | | | | | | | | | | | | | | | | | | | | | | | | | | | | | | | | | | | | | | | | | | | | | | | | | | | | | | | | | | | | | | | | | | | | | | | | | | | | | | | | | | | | | | |  |
|  | | | | | | | | | Muscle events | | | | | | | | | | | | | | | | | | | | | | | | | | | | | | | | Sensory events | | | | | | | | | | | | | Infectious events | | | | | | | | | | | | | | | | | | | | | | | | | | | | | | | | | | | | | | | | |
|  | | | | | | | | | Myalgia & muscle cramps | | | | | Myotoxicity | | | | Myalgia | | | | | Muscle cramps | | | | Elevated CPK | | | | | Muscle weakness | | | | **Any muscle event** | | | | | Dysthesia in legs | | | | Paraesthesia | | | | | **Any sensory event** | | | | Urinary tract infection | | | | | Parotiditis | | | | Shingles | | | | | Upper respiratory tract infection | | | | Nasopharyngitis | | | | | Sinus congestion | | | | Pneumonitis | | | | | Any serious infection event | | | | **Any infectious event** | | | | |
|  | | | | |  | | | | Colchicine | | Comparator | | | Colchicine | | Comparator | | Colchicine | | Comparator | | | Colchicine | | Comparator | | Colchicine | | Comparator | | | Colchicine | | Comparator | | **Colchicine** | | **Comparator** | | | Colchicine | | Comparator | | Colchicine | | Comparator | | | **Colchicine** | | **Comparator** | | Colchicine | | Comparator | | | Colchicine | | Comparator | | Colchicine | | Comparator | | | Colchicine | | Comparator | | Colchicine | | Comparator | | | Colchicine | | Comparator | | Colchicine | | Comparator | | | Colchicine | | Comparator | | **Colchicine** | | **Comparator** | | |
| Aran 2011 [[24](#_ENREF_24)] | | | | |  | | | | NR | | NR | | | NR | | NR | | NR | | NR | | | NR | | NR | | NR | | NR | | | NR | | NR | | **NR** | | **NR** | | | NR | | NR | | NR | | NR | | | **NR** | | **NR** | | NR | | NR | | | NR | | NR | | NR | | NR | | | NR | | NR | | NR | | NR | | | NR | | NR | | NR | | NR | | | NR | | NR | | **NR** | | **NR** | | |
| Batezzati 2001 [[6](#_ENREF_6)] | | | | |  | | | | NR | | NR | | | NR | | NR | | NR | | NR | | | NR | | NR | | NR | | NR | | | NR | | NR | | **NR** | | **NR** | | | NR | | NR | | NR | | NR | | | **NR** | | **NR** | | NR | | NR | | | NR | | NR | | NR | | NR | | | NR | | NR | | NR | | NR | | | NR | | NR | | NR | | NR | | | NR | | NR | | **NR** | | **NR** | | |
| Bessissow 2018 [[36](#_ENREF_36)] | | | | |  | | | | NR | | NR | | | NR | | NR | | NR | | NR | | | NR | | NR | | NR | | NR | | | NR | | NR | | NR | | NR | | | NR | | NR | | NR | | NR | | | NR | | NR | | NR | | NR | | | NR | | NR | | NR | | NR | | | NR | | NR | | NR | | NR | | | NR | | NR | | 0 | | 1 | | | NR | | NR | | **6** | | **8** | | |
| Borstad 2004 [[20](#_ENREF_20)] | | | | |  | | | | NR | | NR | | | NR | | NR | | NR | | NR | | | NR | | NR | | NR | | NR | | | NR | | NR | | **NR** | | **NR** | | | NR | | NR | | NR | | NR | | | **NR** | | **NR** | | NR | | NR | | | NR | | NR | | NR | | NR | | | NR | | NR | | NR | | NR | | | NR | | NR | | NR | | NR | | | NR | | NR | | **NR** | | **NR** | | |
| Cohen 1991 [[33](#_ENREF_33)] | | | | |  | | | | NR | | NR | | | NR | | NR | | NR | | NR | | | NR | | NR | | NR | | NR | | | NR | | NR | | **NR** | | **NR** | | | NR | | NR | | NR | | NR | | | **NR** | | **NR** | | NR | | NR | | | NR | | NR | | NR | | NR | | | NR | | NR | | NR | | NR | | | NR | | NR | | NR | | NR | | | NR | | NR | | **NR** | | **NR** | | |
| Cortez-Pinto 2002 [[7](#_ENREF_7)] | | | | |  | | | | NR | | NR | | | NR | | NR | | NR | | NR | | | NR | | NR | | NR | | NR | | | NR | | NR | | **NR** | | **NR** | | | NR | | NR | | NR | | NR | | | **NR** | | **NR** | | NR | | NR | | | NR | | NR | | NR | | NR | | | NR | | NR | | NR | | NR | | | NR | | NR | | NR | | NR | | | NR | | NR | | **NR** | | **NR** | | |
| Das 2002 [[25](#_ENREF_25)] | | | | |  | | | | NR | | NR | | | NR | | NR | | NR | | NR | | | NR | | NR | | NR | | NR | | | NR | | NR | | **NR** | | **NR** | | | NR | | NR | | NR | | NR | | | **NR** | | **NR** | | NR | | NR | | | NR | | NR | | NR | | NR | | | NR | | NR | | NR | | NR | | | NR | | NR | | NR | | NR | | | NR | | NR | | **NR** | | **NR** | | |
| Davatchi 2009 [[27](#_ENREF_27)] | | | | |  | | | | NR | | NR | | | NR | | NR | | NR | | NR | | | NR | | NR | | NR | | NR | | | NR | | NR | | **NR** | | **NR** | | | 0 | | 1 | | NR | | NR | | | **0** | | **1** | | 1 | | 0 | | | 0 | | 1 | | 0 | | 1 | | | NR | | NR | | NR | | NR | | | NR | | NR | | NR | | NR | | | NR | | NR | | **1** | | **1** | | |
| Deftereos 2013 [[34](#_ENREF_34)] | | | | |  | | | | 15 | | 10 | | | NR | | NR | | NR | | NR | | | NR | | NR | | NR | | NR | | | NR | | NR | | **15** | | **10** | | | NR | | NR | | NR | | NR | | | **NR** | | **NR** | | NR | | NR | | | NR | | NR | | NR | | NR | | | NR | | NR | | NR | | NR | | | NR | | NR | | NR | | NR | | | NR | | NR | | **NR** | | **NR** | | |
| Demidowich 2019 [[35](#_ENREF_35)] | | | | |  | | | | NR | | NR | | | NR | | NR | | NR | | NR | | | NR | | NR | | 4 | | 6 | | | NR | | NR | | **4** | | **6** | | | NR | | NR | | NR | | NR | | | NR | | NR | | NR | | NR | | | NR | | NR | | NR | | NR | | | 6 | | 11 | | NR | | NR | | | NR | | NR | | NR | | NR | | | NR | | NR | | **6** | | **11** | | |
| Dinarello 1974 [[12](#_ENREF_12)] | | | | |  | | | | NR | | NR | | | NR | | NR | | NR | | NR | | | NR | | NR | | NR | | NR | | | NR | | NR | | **NR** | | **NR** | | | NR | | NR | | NR | | NR | | | **NR** | | **NR** | | NR | | NR | | | NR | | NR | | NR | | NR | | | NR | | NR | | NR | | NR | | | NR | | NR | | NR | | NR | | | NR | | NR | | **NR** | | **NR** | | |
| Fish 1997 [[38](#_ENREF_38)] | | | | |  | | | | NR | | NR | | | NR | | NR | | NR | | NR | | | NR | | NR | | NR | | NR | | | NR | | NR | | **NR** | | **NR** | | | NR | | NR | | NR | | NR | | | **NR** | | **NR** | | NR | | NR | | | NR | | NR | | NR | | NR | | | NR | | NR | | NR | | NR | | | NR | | NR | | NR | | NR | | | NR | | NR | | **NR** | | **NR** | | |
| Imazio 2010 [[31](#_ENREF_31)] | | | | |  | | | | NR | | NR | | | 0 | | 1 | | NR | | NR | | | NR | | NR | | NR | | NR | | | NR | | NR | | **0** | | **1** | | | NR | | NR | | NR | | NR | | | **NR** | | **NR** | | NR | | NR | | | NR | | NR | | NR | | NR | | | NR | | NR | | NR | | NR | | | NR | | NR | | NR | | NR | | | NR | | NR | | **NR** | | **NR** | | |
| Imazio 2011[[16](#_ENREF_16)] | | | | |  | | | | NR | | NR | | | 0 | | 0 | | NR | | NR | | | NR | | NR | | NR | | NR | | | NR | | NR | | **0** | | **0** | | | NR | | NR | | NR | | NR | | | **NR** | | **NR** | | NR | | NR | | | NR | | NR | | NR | | NR | | | NR | | NR | | NR | | NR | | | NR | | NR | | NR | | NR | | | NR | | NR | | **NR** | | **NR** | | |
| Imazio 2013 [[17](#_ENREF_17)] | | | | |  | | | | NR | | NR | | | 0 | | 0 | | NR | | NR | | | NR | | NR | | NR | | NR | | | NR | | NR | | **0** | | **0** | | | NR | | NR | | NR | | NR | | | **NR** | | **NR** | | NR | | NR | | | NR | | NR | | NR | | NR | | | NR | | NR | | NR | | NR | | | NR | | NR | | NR | | NR | | | NR | | NR | | **NR** | | **NR** | | |
| Imazio 2014a [[32](#_ENREF_32)] | | | | |  | | | | NR | | NR | | | 0 | | 0 | | NR | | NR | | | NR | | NR | | NR | | NR | | | NR | | NR | | **0** | | **0** | | | NR | | NR | | NR | | NR | | | **NR** | | **NR** | | NR | | NR | | | NR | | NR | | NR | | NR | | | NR | | NR | | NR | | NR | | | NR | | NR | | NR | | NR | | | NR | | NR | | **NR** | | **NR** | | |
| Imazio 2014b [[18](#_ENREF_18)] | | | | |  | | | | NR | | NR | | | 1 | | 0 | | NR | | NR | | | NR | | NR | | NR | | NR | | | NR | | NR | | **1** | | **0** | | | NR | | NR | | NR | | NR | | | **NR** | | **NR** | | NR | | NR | | | NR | | NR | | NR | | NR | | | NR | | NR | | NR | | NR | | | NR | | NR | | NR | | NR | | | NR | | NR | | **NR** | | **NR** | | |
| Kaplan 1986 [[8](#_ENREF_8)] | | | | |  | | | | NR | | NR | | | NR | | NR | | NR | | NR | | | NR | | NR | | NR | | NR | | | NR | | NR | | **NR** | | **NR** | | | NR | | NR | | NR | | NR | | | **NR** | | **NR** | | NR | | NR | | | NR | | NR | | NR | | NR | | | NR | | NR | | NR | | NR | | | NR | | NR | | NR | | NR | | | NR | | NR | | **NR** | | **NR** | | |
| Kershenobich 1979 [[9](#_ENREF_9)] | | | | |  | | | | NR | | NR | | | NR | | NR | | NR | | NR | | | NR | | NR | | NR | | NR | | | NR | | NR | | **NR** | | **NR** | | | NR | | NR | | NR | | NR | | | **NR** | | **NR** | | NR | | NR | | | NR | | NR | | NR | | NR | | | NR | | NR | | NR | | NR | | | NR | | NR | | NR | | NR | | | NR | | NR | | **NR** | | **NR** | | |
| Kershenobich 1988 [[10](#_ENREF_10)] | | | | |  | | | | NR | | NR | | | NR | | NR | | NR | | NR | | | NR | | NR | | NR | | NR | | | NR | | NR | | **NR** | | **NR** | | | NR | | NR | | NR | | NR | | | **NR** | | **NR** | | NR | | NR | | | NR | | NR | | NR | | NR | | | NR | | NR | | NR | | NR | | | NR | | NR | | NR | | NR | | | NR | | NR | | **NR** | | **NR** | | |
| Leung 2018 [[26](#_ENREF_26)] | | | | |  | | | | NR | | NR | | | NR | | NR | | 12 | | 6 | | | 7 | | 8 | | 9 | | 3 | | | NR | | NR | | **12** | | **8** | | | NR | | NR | | 2 | | 2 | | | **2** | | **2** | | 0 | | 1 | | | NR | | NR | | NR | | NR | | | 17 | | 10 | | NR | | NR | | | NR | | NR | | NR | | NR | | | NR | | NR | | **17** | | **10** | | |
| Liebenburg 2016[[19](#_ENREF_19)] | | | | |  | | | | NR | | NR | | | NR | | NR | | NR | | NR | | | NR | | NR | | NR | | NR | | | NR | | NR | | **NR** | | **NR** | | | NR | | NR | | NR | | NR | | | **NR** | | **NR** | | NR | | NR | | | NR | | NR | | NR | | NR | | | NR | | NR | | NR | | NR | | | NR | | NR | | NR | | NR | | | NR | | NR | | **NR** | | **NR** | | |
| Masuda 1989 [[28](#_ENREF_28)] | | | | |  | | | | NR | | NR | | | NR | | NR | | NR | | NR | | | NR | | NR | | NR | | NR | | | NR | | NR | | **NR** | | **NR** | | | NR | | NR | | NR | | NR | | | **NR** | | **NR** | | NR | | NR | | | NR | | NR | | NR | | NR | | | NR | | NR | | NR | | NR | | | NR | | NR | | NR | | NR | | | NR | | NR | | **NR** | | **NR** | | |
| McKendry 1993 [[13](#_ENREF_13)] | | | | |  | | | | NR | | NR | | | NR | | NR | | NR | | NR | | | NR | | NR | | 5 | | 4 | | | 0 | | 0 | | **5** | | **4** | | | NR | | NR | | NR | | NR | | | **NR** | | **NR** | | NR | | NR | | | NR | | NR | | NR | | NR | | | NR | | NR | | NR | | NR | | | NR | | NR | | NR | | NR | | | NR | | NR | | **NR** | | **NR** | | |
| Olsson 1995 [[11](#_ENREF_11)] | | | | |  | | | | NR | | NR | | | NR | | NR | | NR | | NR | | | NR | | NR | | NR | | NR | | | NR | | NR | | **NR** | | **NR** | | | NR | | NR | | NR | | NR | | | **NR** | | **NR** | | NR | | NR | | | NR | | NR | | NR | | NR | | | NR | | NR | | NR | | NR | | | NR | | NR | | NR | | NR | | | NR | | NR | | **NR** | | **NR** | | |
| Pakfetrat 2010 [[39](#_ENREF_39)] | | | | |  | | | | NR | | NR | | | NR | | NR | | NR | | NR | | | NR | | NR | | NR | | NR | | | NR | | NR | | **NR** | | **NR** | | | NR | | NR | | NR | | NR | | | **NR** | | **NR** | | NR | | NR | | | NR | | NR | | NR | | NR | | | NR | | NR | | NR | | NR | | | NR | | NR | | NR | | NR | | | NR | | NR | | **NR** | | **NR** | | |
| Paulus 1974 [[21](#_ENREF_21)] | | | | |  | | | | NR | | NR | | | NR | | NR | | NR | | NR | | | NR | | NR | | NR | | NR | | | NR | | NR | | **NR** | | **NR** | | | NR | | NR | | NR | | NR | | | **NR** | | **NR** | | NR | | NR | | | NR | | NR | | NR | | NR | | | NR | | NR | | NR | | NR | | | NR | | NR | | NR | | NR | | | NR | | NR | | **11** | | **5** | | |
| Roche 1995 [[14](#_ENREF_14)] | | | | |  | | | | NR | | NR | | | NR | | NR | | NR | | NR | | | NR | | NR | | NR | | NR | | | NR | | NR | | **NR** | | **NR** | | | NR | | NR | | NR | | NR | | | **NR** | | **NR** | | NR | | NR | | | NR | | NR | | NR | | NR | | | NR | | NR | | NR | | NR | | | NR | | NR | | NR | | NR | | | NR | | NR | | **NR** | | **NR** | | |
| Schlesinger 2011[[15](#_ENREF_15)] | | | | |  | | | | NR | | NR | | | NR | | NR | | NR | | NR | | | NR | | NR | | NR | | NR | | | NR | | NR | | **NR** | | **NR** | | | NR | | NR | | NR | | NR | | | **NR** | | **NR** | | NR | | NR | | | NR | | NR | | NR | | NR | | | 4 | | 12 | | 1 | | 12 | | | 1 | | 3 | | NR | | NR | | | 0 | | 4 | | **13** | | **58** | | |
| Schnebel 1988 [[40](#_ENREF_40)] | | | | |  | | | | NR | | NR | | | NR | | NR | | NR | | NR | | | NR | | NR | | NR | | NR | | | NR | | NR | | **NR** | | **NR** | | | NR | | NR | | NR | | NR | | | **NR** | | **NR** | | NR | | NR | | | NR | | NR | | NR | | NR | | | NR | | NR | | NR | | NR | | | NR | | NR | | NR | | NR | | | NR | | NR | | **NR** | | **NR** | | |
| Seideman 1987 [[30](#_ENREF_30)] | | | | |  | | | | NR | | NR | | | NR | | NR | | NR | | NR | | | NR | | NR | | NR | | NR | | | NR | | NR | | **NR** | | **NR** | | | NR | | NR | | NR | | NR | | | **NR** | | **NR** | | NR | | NR | | | NR | | NR | | NR | | NR | | | NR | | NR | | NR | | NR | | | NR | | NR | | NR | | NR | | | NR | | NR | | **NR** | | **NR** | | |
| Tardif 2019 [[37](#_ENREF_37)] | | | | |  | | | | NR | | NR | | | NR | | NR | | NR | | NR | | | NR | | NR | | NR | | NR | | | NR | | NR | | NR | | NR | | | NR | | NR | | NR | | NR | | | NR | | NR | | NR | | NR | | | NR | | NR | | NR | | NR | | | NR | | NR | | NR | | NR | | | NR | | NR | | 21 | | 9 | | | 51 | | 38 | | **51** | | **38** | | |
| Terkeltaub 2010 [[22](#_ENREF_22)] | | | | | Low dose | | | | NR | | NR | | | NR | | NR | | NR | | NR | | | NR | | NR | | NR | | NR | | | NR | | NR | | **NR** | | **NR** | | | NR | | NR | | NR | | NR | | | **NR** | | **NR** | | NR | | NR | | | NR | | NR | | NR | | NR | | | NR | | NR | | NR | | NR | | | NR | | NR | | NR | | NR | | | NR | | NR | | **NR** | | **NR** | | |
|  |  |  |  |  | High dose | | | | NR | | NR | | | NR | | NR | | NR | | NR | | | NR | | NR | | NR | | NR | | | NR | | NR | | **NR** | | **NR** | | | NR | | NR | | NR | | NR | | | **NR** | | **NR** | | NR | | NR | | | NR | | NR | | NR | | NR | | | NR | | NR | | NR | | NR | | | NR | | NR | | NR | | NR | | | NR | | NR | | **NR** | | **NR** | | |
|  |  |  |  |  | Total | | | | NR | | NR | | | NR | | NR | | NR | | NR | | | NR | | NR | | NR | | NR | | | NR | | NR | | **NR** | | **NR** | | | NR | | NR | | NR | | NR | | | **NR** | | **NR** | | NR | | NR | | | NR | | NR | | NR | | NR | | | NR | | NR | | NR | | NR | | | NR | | NR | | NR | | NR | | | NR | | NR | | **NR** | | **NR** | | |
| Wang 2014 [[23](#_ENREF_23)] | | | | |  | | | | NR | | NR | | | NR | | NR | | NR | | NR | | | NR | | NR | | NR | | NR | | | NR | | NR | | **NR** | | **NR** | | | NR | | NR | | NR | | NR | | | **NR** | | **NR** | | NR | | NR | | | NR | | NR | | NR | | NR | | | NR | | NR | | NR | | NR | | | NR | | NR | | NR | | NR | | | NR | | NR | | **NR** | | **NR** | | |
| Yurdakul 2001 [[29](#_ENREF_29)] | | | | |  | | | | NR | | NR | | | NR | | NR | | NR | | NR | | | NR | | NR | | NR | | NR | | | NR | | NR | | **NR** | | **NR** | | | NR | | NR | | NR | | NR | | | **NR** | | **NR** | | NR | | NR | | | NR | | NR | | NR | | NR | | | NR | | NR | | NR | | NR | | | NR | | NR | | NR | | NR | | | NR | | NR | | **NR** | | **NR** | | |

| **Supplementary Table 6**. Number of participants in colchicine and comparator groups with miscellaneous adverse events or death | | | | | | | | | | | | | | | | | | | | | | | | | | | | | | | | | | | | | | | |
| --- | --- | --- | --- | --- | --- | --- | --- | --- | --- | --- | --- | --- | --- | --- | --- | --- | --- | --- | --- | --- | --- | --- | --- | --- | --- | --- | --- | --- | --- | --- | --- | --- | --- | --- | --- | --- | --- | --- | --- |
|  | | Alopecia | | Hirsutism | | Pruritus | | Bullous dermatitis | | Rash | | Hematuria | | Dry mouth | | Dizziness | | Vertigo | | Neurologic | | Fatigue | | Headache | | Headache, anxiety & fatigue | | Back pain | | Renal abnormalities | | Insomnia | | Allergic reaction | | Hypertension | | Death | |
|  |  | Colchicine | Comparator | Colchicine | Comparator | Colchicine | Comparator | Colchicine | Comparator | Colchicine | Comparator | Colchicine | Comparator | Colchicine | Comparator | Colchicine | Comparator | Colchicine | Comparator | Colchicine | Comparator | Colchicine | Comparator | Colchicine | Comparator | Colchicine | Comparator | Colchicine | Comparator | Colchicine | Comparator | Colchicine | Comparator | Colchicine | Comparator | Colchicine | Comparator | Colchicine | Comparator |
| Aran 2011 [[24](#_ENREF_24)] |  | NR | NR | NR | NR | NR | NR | NR | NR | NR | NR | NR | NR | NR | NR | NR | NR | NR | NR | NR | NR | NR | NR | NR | NR | NR | NR | NR | NR | NR | NR | NR | NR | NR | NR | NR | NR | NR | NR |
| Batezzati 2001 [[6](#_ENREF_6)] |  | NR | NR | NR | NR | NR | NR | NR | NR | NR | NR | NR | NR | NR | NR | NR | NR | NR | NR | NR | NR | NR | NR | NR | NR | NR | NR | NR | NR | NR | NR | NR | NR | NR | NR | NR | NR | NR | NR |
| Bessissow 2018 [[36](#_ENREF_36)] |  | NR | NR | NR | NR | NR | NR | NR | NR | NR | NR | NR | NR | NR | NR | NR | NR | NR | NR | NR | NR | NR | NR | NR | NR | NR | NR | NR | NR | 0 | 0 | NR | NR | NR | NR | NR | NR | 0 | 0 |
| Borstad 2004 [[20](#_ENREF_20)] |  | NR | NR | NR | NR | NR | NR | NR | NR | NR | NR | NR | NR | NR | NR | NR | NR | NR | NR | NR | NR | NR | NR | NR | NR | NR | NR | NR | NR | NR | NR | NR | NR | NR | NR | NR | NR | NR | NR |
| Cohen 1991 [[33](#_ENREF_33)] |  | NR | NR | NR | NR | NR | NR | NR | NR | NR | NR | NR | NR | NR | NR | NR | NR | NR | NR | NR | NR | NR | NR | NR | NR | NR | NR | NR | NR | NR | NR | NR | NR | NR | NR | NR | NR | NR | NR |
| Cortez-Pinto 2002 [[7](#_ENREF_7)] |  | NR | NR | NR | NR | NR | NR | NR | NR | NR | NR | NR | NR | NR | NR | NR | NR | NR | NR | NR | NR | NR | NR | NR | NR | NR | NR | NR | NR | NR | NR | NR | NR | NR | NR | NR | NR | NR | NR |
| Das 2002 [[25](#_ENREF_25)] |  | NR | NR | NR | NR | NR | NR | NR | NR | NR | NR | NR | NR | NR | NR | NR | NR | NR | NR | NR | NR | NR | NR | NR | NR | NR | NR | NR | NR | NR | NR | NR | NR | NR | NR | NR | NR | 0 | 0 |
| Davatchi 2009 [[27](#_ENREF_27)] |  | NR | NR | NR | NR | NR | NR | NR | NR | NR | NR | 0 | 1 | NR | NR | NR | NR | NR | NR | NR | NR | NR | NR | NR | NR | NR | NR | NR | NR | NR | NR | NR | NR | NR | NR | NR | NR | NR | NR |
| Deftereos 2013 [[34](#_ENREF_34)] |  | 1 | 1 | NR | NR | NR | NR | NR | NR | NR | NR | NR | NR | NR | NR | NR | NR | NR | NR | NR | NR | NR | NR | NR | NR | NR | NR | NR | NR | NR | NR | NR | NR | NR | NR | NR | NR | NR | NR |
| Demidowich 2019 [[35](#_ENREF_35)] |  | NR | NR | NR | NR | NR | NR | NR | NR | 2 | 2 | NR | NR | NR | NR | NR | NR | NR | NR | 3 | 3 | 6 | 5 | 3 | 4 | NR | NR | NR | NR | 0 | 0 | NR | NR | NR | NR | NR | NR | NR | NR |
| Dinarello 1974 [[12](#_ENREF_12)] |  | NR | NR | NR | NR | NR | NR | NR | NR | NR | NR | NR | NR | NR | NR | NR | NR | NR | NR | NR | NR | NR | NR | NR | NR | NR | NR | NR | NR | 0 | 0 | NR | NR | NR | NR | NR | NR | NR | NR |
| Fish 1997 [[38](#_ENREF_38)] |  | NR | NR | NR | NR | NR | NR | NR | NR | NR | NR | NR | NR | NR | NR | NR | NR | NR | NR | NR | NR | NR | NR | NR | NR | NR | NR | NR | NR | NR | NR | NR | NR | NR | NR | NR | NR | NR | NR |
| Imazio 2010 [[31](#_ENREF_31)] |  | 0 | 0 | NR | NR | NR | NR | NR | NR | NR | NR | NR | NR | NR | NR | NR | NR | NR | NR | NR | NR | NR | NR | NR | NR | NR | NR | NR | NR | NR | NR | NR | NR | NR | NR | NR | NR | NR | NR |
| Imazio 2011[[16](#_ENREF_16)] |  | 0 | 0 | NR | NR | NR | NR | NR | NR | NR | NR | NR | NR | NR | NR | NR | NR | NR | NR | NR | NR | NR | NR | NR | NR | NR | NR | NR | NR | NR | NR | NR | NR | NR | NR | NR | NR | NR | NR |
| Imazio 2013 [[17](#_ENREF_17)] |  | 1 | 1 | NR | NR | NR | NR | NR | NR | NR | NR | NR | NR | NR | NR | NR | NR | NR | NR | NR | NR | NR | NR | NR | NR | NR | NR | NR | NR | NR | NR | NR | NR | NR | NR | NR | NR | NR | NR |
| Imazio 2014a [[32](#_ENREF_32)] |  | 0 | 0 | NR | NR | NR | NR | NR | NR | NR | NR | NR | NR | NR | NR | NR | NR | NR | NR | NR | NR | NR | NR | NR | NR | NR | NR | NR | NR | NR | NR | NR | NR | NR | NR | NR | NR | NR | NR |
| Imazio 2014b [[18](#_ENREF_18)] |  | 1 | 0 | NR | NR | NR | NR | NR | NR | NR | NR | NR | NR | NR | NR | NR | NR | NR | NR | NR | NR | NR | NR | NR | NR | NR | NR | NR | NR | NR | NR | NR | NR | NR | NR | NR | NR | NR | NR |
| Kaplan 1986 [[8](#_ENREF_8)] |  | NR | NR | NR | NR | NR | NR | NR | NR | NR | NR | NR | NR | NR | NR | NR | NR | NR | NR | NR | NR | NR | NR | NR | NR | 1 | 1 | NR | NR | NR | NR | NR | NR | NR | NR | NR | NR | NR | NR |
| Kershenobich 1979 [[9](#_ENREF_9)] |  | NR | NR | NR | NR | NR | NR | NR | NR | NR | NR | NR | NR | NR | NR | NR | NR | NR | NR | NR | NR | NR | NR | NR | NR | NR | NR | NR | NR | NR | NR | NR | NR | NR | NR | NR | NR | NR | NR |
| Kershenobich 1988 [[10](#_ENREF_10)] |  | NR | NR | NR | NR | NR | NR | NR | NR | NR | NR | NR | NR | NR | NR | NR | NR | NR | NR | NR | NR | NR | NR | NR | NR | NR | NR | NR | NR | NR | NR | NR | NR | NR | NR | NR | NR | NR | NR |
| Leung 2018 [[26](#_ENREF_26)] |  | NR | NR | NR | NR | 0 | 1 | 1 | 0 | NR | NR | NR | NR | 1 | 1 | 1 | 2 | NR | NR | NR | NR | NR | NR | 2 | 1 | NR | NR | NR | NR | NR | NR | 0 | 1 | 1 | 1 | NR | NR | 0 | 0 |
| Liebenburg 2016[[19](#_ENREF_19)] |  | NR | NR | NR | NR | NR | NR | NR | NR | NR | NR | NR | NR | NR | NR | NR | NR | NR | NR | NR | NR | NR | NR | NR | NR | NR | NR | NR | NR | NR | NR | NR | NR | NR | NR | NR | NR | NR | NR |
| Masuda 1989 [[28](#_ENREF_28)] |  | NR | NR | 2 | 23 | NR | NR | NR | NR | NR | NR | NR | NR | NR | NR | NR | NR | NR | NR | NR | NR | NR | NR | NR | NR | NR | NR | NR | NR | NR | NR | 2 | 11 | NR | NR | NR | NR | NR | NR |
| McKendry 1993 [[13](#_ENREF_13)] |  | NR | NR | NR | NR | NR | NR | NR | NR | NR | NR | NR | NR | NR | NR | NR | NR | NR | NR | NR | NR | NR | NR | NR | NR | NR | NR | NR | NR | NR | NR | NR | NR | NR | NR | NR | NR | NR | NR |
| Olsson 1995 [[11](#_ENREF_11)] |  | NR | NR | NR | NR | NR | NR | NR | NR | NR | NR | NR | NR | NR | NR | NR | NR | NR | NR | NR | NR | NR | NR | NR | NR | NR | NR | NR | NR | NR | NR | NR | NR | NR | NR | NR | NR | NR | NR |
| Pakfetrat 2010 [[39](#_ENREF_39)] |  | NR | NR | NR | NR | NR | NR | NR | NR | NR | NR | NR | NR | NR | NR | NR | NR | 3 | 0 | NR | NR | NR | NR | 1 | 1 | NR | NR | NR | NR | NR | NR | NR | NR | NR | NR | 0 | 1 | NR | NR |
| Paulus 1974 [[21](#_ENREF_21)] |  | 1 | 0 | NR | NR | NR | NR | NR | NR | NR | NR | NR | NR | NR | NR | NR | NR | NR | NR | NR | NR | NR | NR | NR | NR | NR | NR | NR | NR | NR | NR | NR | NR | NR | NR | NR | NR | NR | NR |
| Roche 1995 [[14](#_ENREF_14)] |  | NR | NR | NR | NR | NR | NR | NR | NR | NR | NR | NR | NR | NR | NR | NR | NR | NR | NR | NR | NR | NR | NR | NR | NR | NR | NR | NR | NR | NR | NR | NR | NR | NR | NR | NR | NR | NR | NR |
| Schlesinger 2011[[15](#_ENREF_15)] |  | NR | NR | NR | NR | NR | NR | NR | NR | NR | NR | NR | NR | NR | NR | NR | NR | NR | NR | NR | NR | NR | NR | 6 | 19 | NR | NR | 4 | 10 | NR | NR | NR | NR | NR | NR | 1 | 21 | NR | NR |
| Schnebel 1988 [[40](#_ENREF_40)] |  | NR | NR | NR | NR | NR | NR | NR | NR | NR | 1 | 5 | NR | NR | NR | NR | NR | NR | NR | NR | NR | NR | NR | NR | NR | NR | NR | NR | NR | NR | NR | NR | NR | NR | NR | NR | NR | NR | NR |
| Seideman 1987 [[30](#_ENREF_30)] |  | NR | NR | NR | NR | NR | NR | NR | NR | NR | NR | NR | NR | NR | NR | NR | NR | NR | NR | NR | NR | NR | NR | NR | NR | NR | NR | NR | NR | NR | NR | NR | NR | NR | NR | NR | NR | NR | NR |
| Tardif 2019 [[37](#_ENREF_37)] |  | NR | NR | NR | NR | NR | NR | NR | NR | NR | NR | NR | NR | NR | NR | NR | NR | NR | NR | NR | NR | NR | NR | NR | NR | NR | NR | NR | NR | NR | NR | NR | NR | NR | NR | NR | NR | NR | NR |
| Terkeltaub 2010 [[22](#_ENREF_22)] | Low dose | NR | NR | NR | NR | NR | NR | NR | NR | NR | NR | NR | NR | NR | NR | NR | NR | NR | NR | NR | NR | NR | NR | NR | NR | NR | NR | NR | NR | NR | NR | NR | NR | NR | NR | NR | NR | 0 | 0 |
|  | High dose | NR | NR | NR | NR | NR | NR | NR | NR | NR | NR | NR | NR | NR | NR | NR | NR | NR | NR | NR | NR | NR | NR | NR | NR | NR | NR | NR | NR | NR | NR | NR | NR | NR | NR | NR | NR | 0 | 0 |
|  | Total | NR | NR | NR | NR | NR | NR | NR | NR | NR | NR | NR | NR | NR | NR | NR | NR | NR | NR | NR | NR | NR | NR | NR | NR | NR | NR | NR | NR | NR | NR | NR | NR | NR | NR | NR | NR | 0 | 0 |
| Wang 2014 [[23](#_ENREF_23)] |  | NR | NR | NR | NR | NR | NR | NR | NR | NR | NR | NR | NR | NR | NR | NR | NR | NR | NR | NR | NR | NR | NR | NR | NR | NR | NR | NR | NR | NR | NR | NR | NR | NR | NR | NR | NR | NR | NR |
| Yurdakul 2001 [[29](#_ENREF_29)] |  | NR | NR | NR | NR | NR | NR | NR | NR | NR | NR | NR | NR | NR | NR | NR | NR | NR | NR | NR | NR | NR | NR | NR | NR | NR | NR | NR | NR | NR | NR | NR | NR | NR | NR | NR | NR | NR | NR |

**
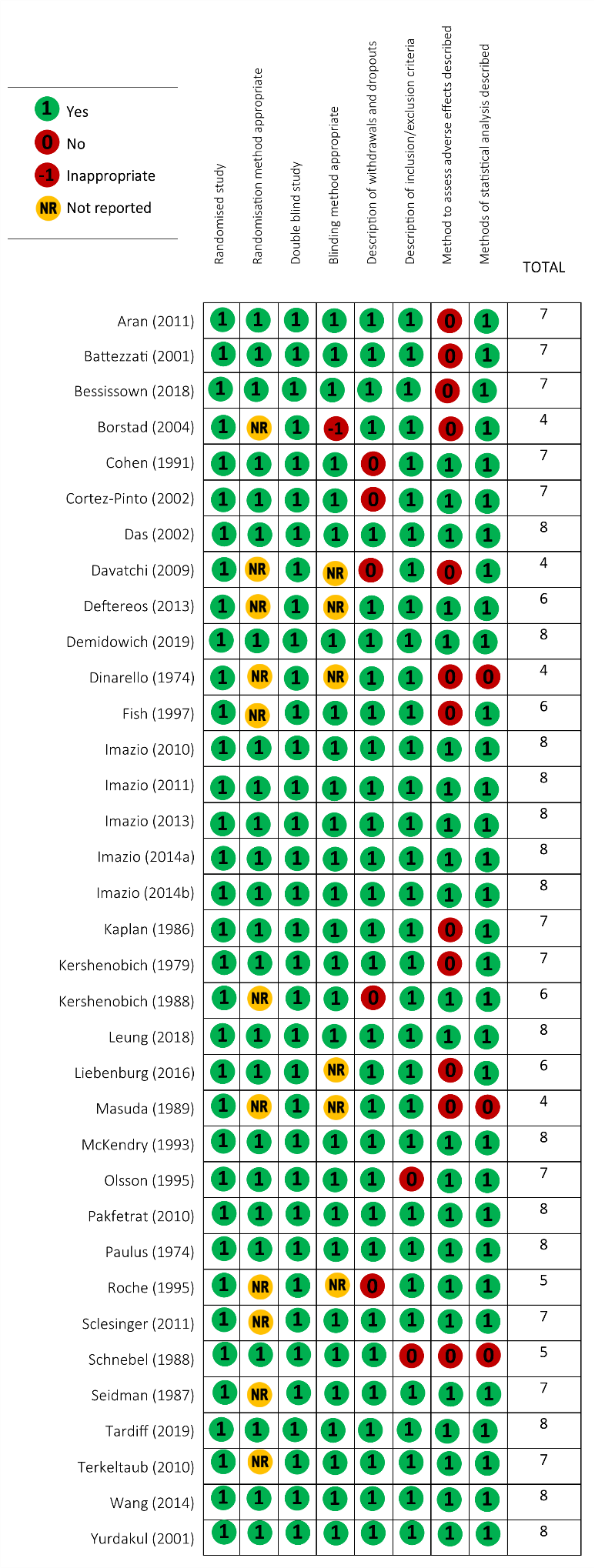
**

**Supplementary Figure 1.** Quality assessment results using the modified-Jadad score


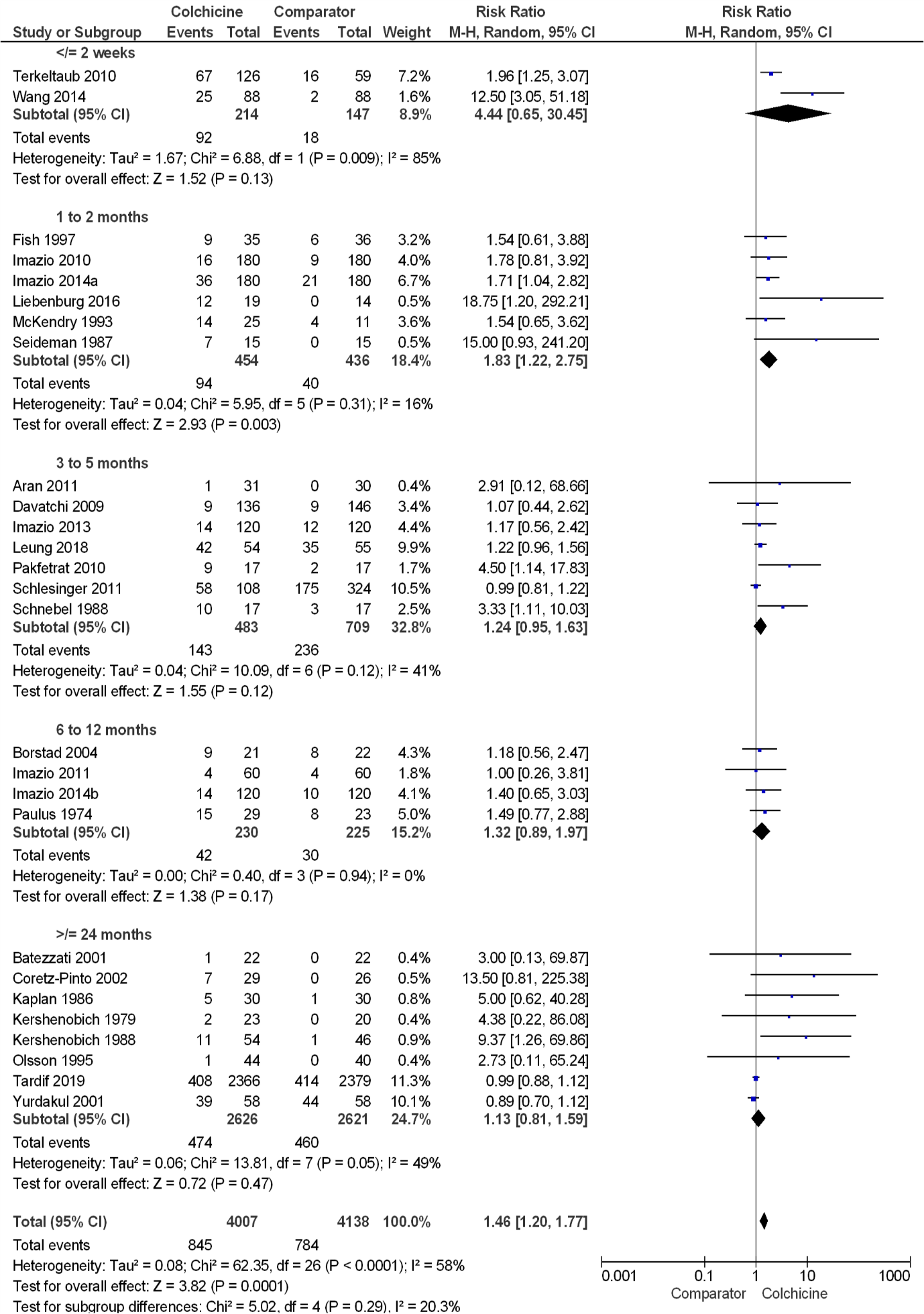


**Supplementary Figure 2.** Forest plot showing estimated relative risk of any adverse event during colchicine use compared to comparator groups across different durations of drug exposure


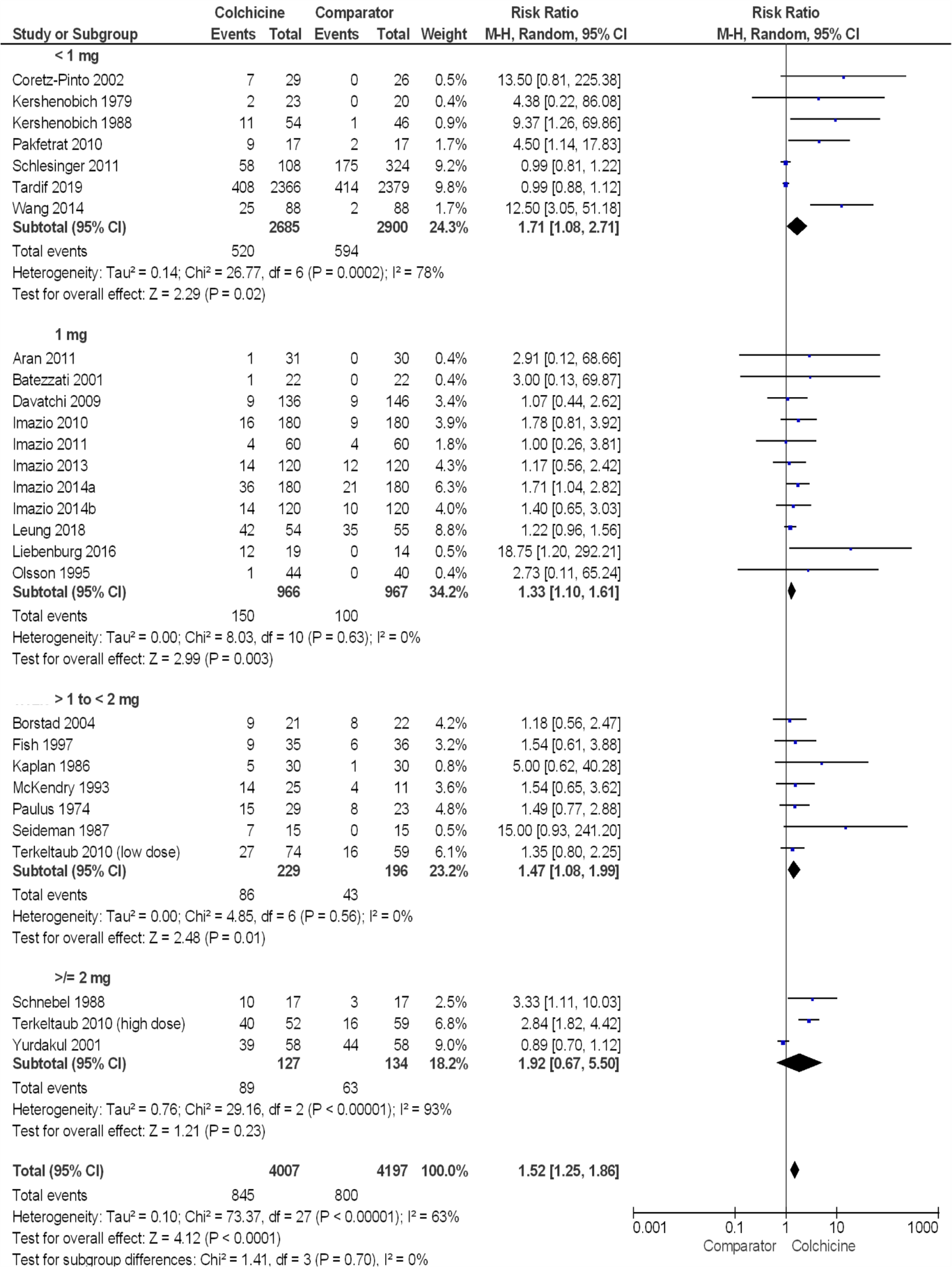


**Supplementary Figure 3.** Forest plot showing estimated relative risk of any adverse event during colchicine use compared to comparator groups across different daily doses of colchicine


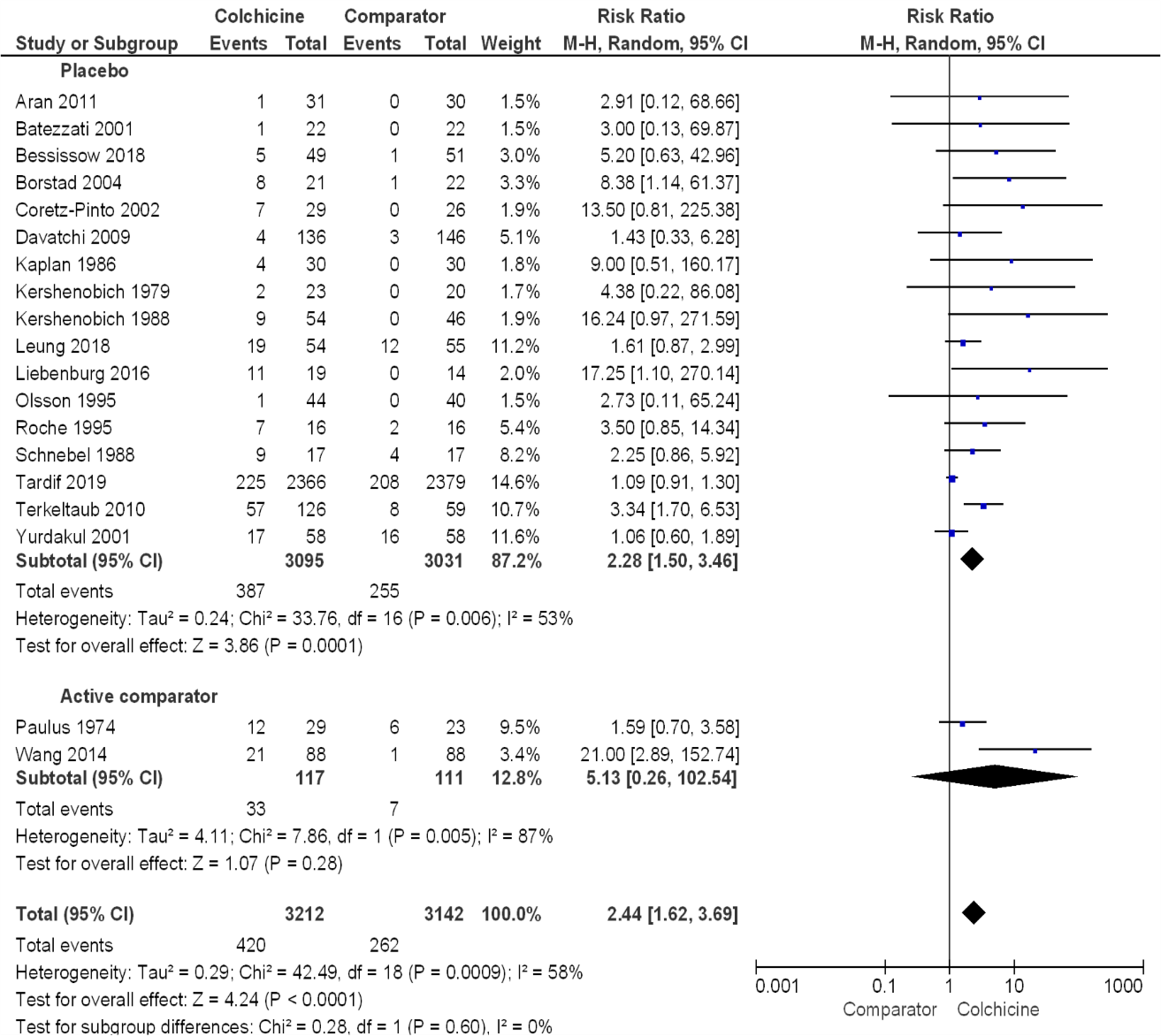


**Supplementary Figure 4.** Forest plot showing estimated relative risk of diarrhoea during colchicine use compared to placebo and active comparator groups


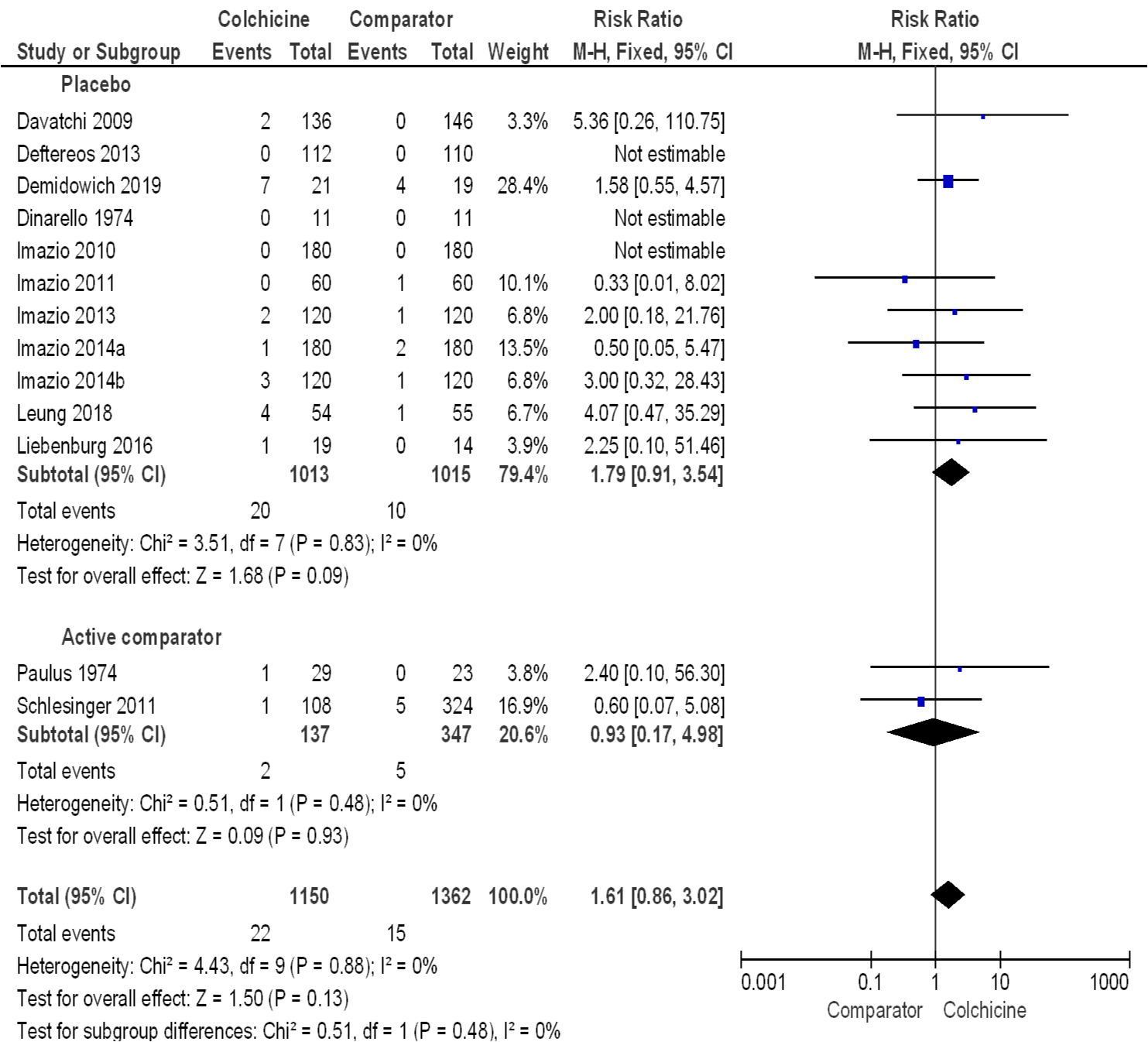


**Supplementary Figure 5.** Forest plot showing estimated relative risk of liver events during colchicine use compared to placebo and active comparator groups


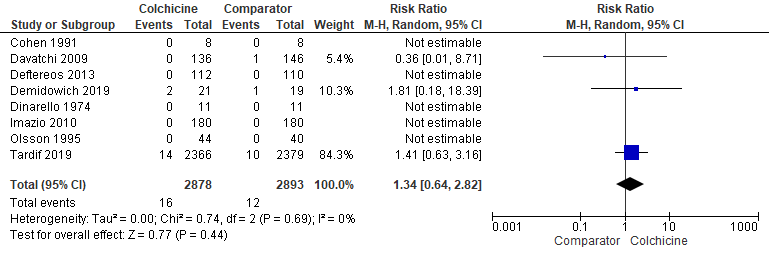


**Supplementary Figure 6.** Forest plot showing estimated relative risk of hematology events during colchicine use compared to placebo (no active comparator studies)


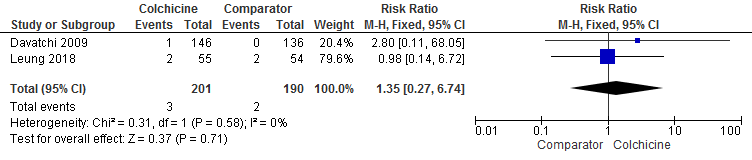


**Supplementary Figure 7.** Forest plot showing estimated relative risk of sensory events during colchicine use compared to placebo (no active comparator studies)


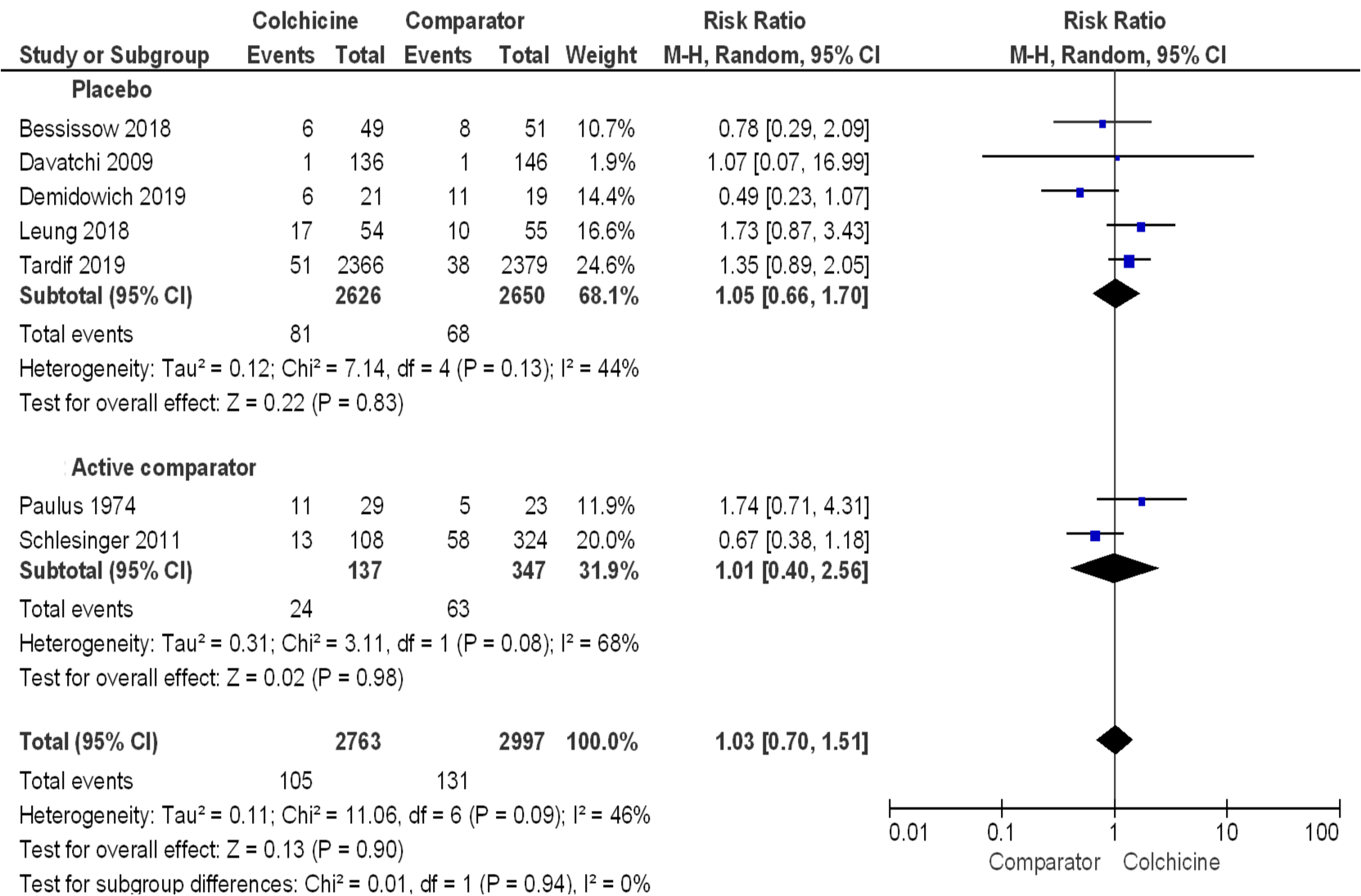


**Supplementary Figure 8.** Forest plot showing estimated relative risk of infectious events during colchicine use compared to placebo and active comparator groups
